# Supplementary material for: Sterol methyltransferases in uncultured bacteria complicate eukaryotic biomarker interpretations
Source: Nat Commun. 2023 Apr 3;14:1859. doi: 10.1038/s41467-023-37552-3 (PMC10070321; doi:10.1038/s41467-023-37552-3)
Supplement: Supplementary file 1 — Supplementary Information [file 41467_2023_37552_MOESM1_ESM.pdf]

**Supplementary Information**

# Sterol methyltransferases in uncultured bacteria complicate eukaryotic biomarker interpretations

Malory O. Brown[a], Babatunde O. Olagunju[b], José-Luis Giner[b], and Paula V. Welander[a]*

[a]Department of Earth System Science, Stanford University, Stanford, CA, 94305, USA

[b]Department of Chemistry, State University of New York-Environmental Science and Forestry, Syracuse, NY, 13210, USA

***corresponding author**: welander@stanford.edu

**Contains:**

Supplementary Figures 1-7

Supplementary Tables 1-5

Supplementary References

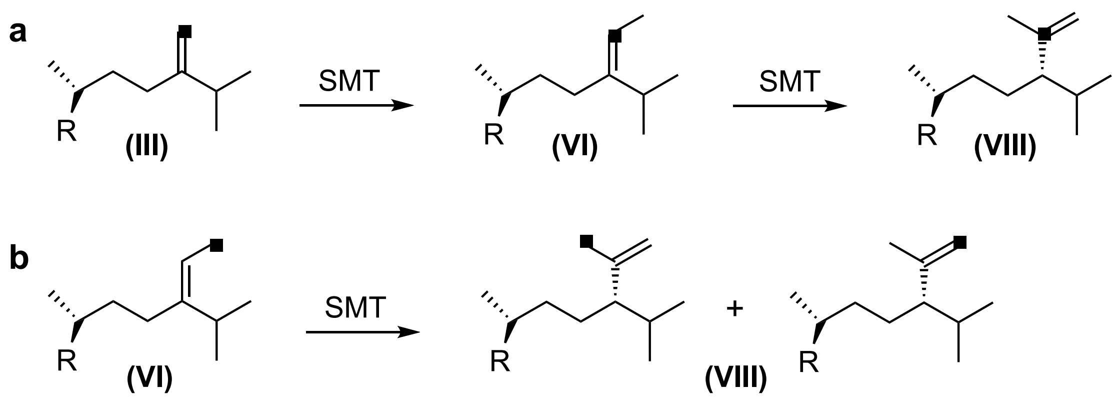


Supplementary Figure 1. **$^{13}$C-labeling experiments with propylating SMTs from an *Aplysina aerophoba* symbiont and a Chlamydiae sp.** Positions of the $^{13}$C labels are shown for the predominant propylation pathway proceeding from 24-methylenecholesterol (III) to isofucosterol (VI) to 24S-24-isopropylcholest-5,25,dienol (VIII). **a** Experiments with [28-$^{13}$C] 24-methylenecholsterol as the substrate. **b** Experiments with [29-$^{13}$C] isofucosterol as the substrate.

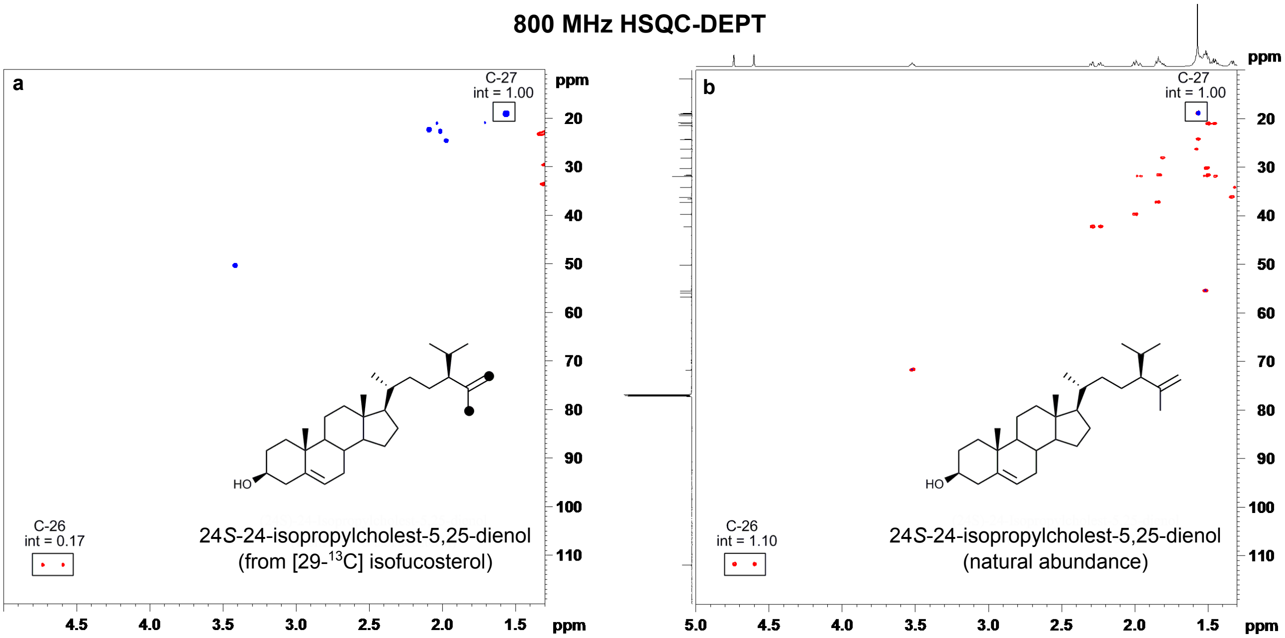


Supplementary Figure 2. **2D NMR (HSQC). a** 24*S*-24-isopropylcholest-5,25,dienol (VIII) from an in vitro reaction performed with [29-$^{13}$C] isofucosterol and a Chlamydiae sp. MAG SMT and **b** naturally abundant 24*S*-24-isopropylcholest-5,25,dienol (VIII). Additional NMR spectra are shown in Supplementary Figures 4-7 and are summarized in Supplementary Table 3.

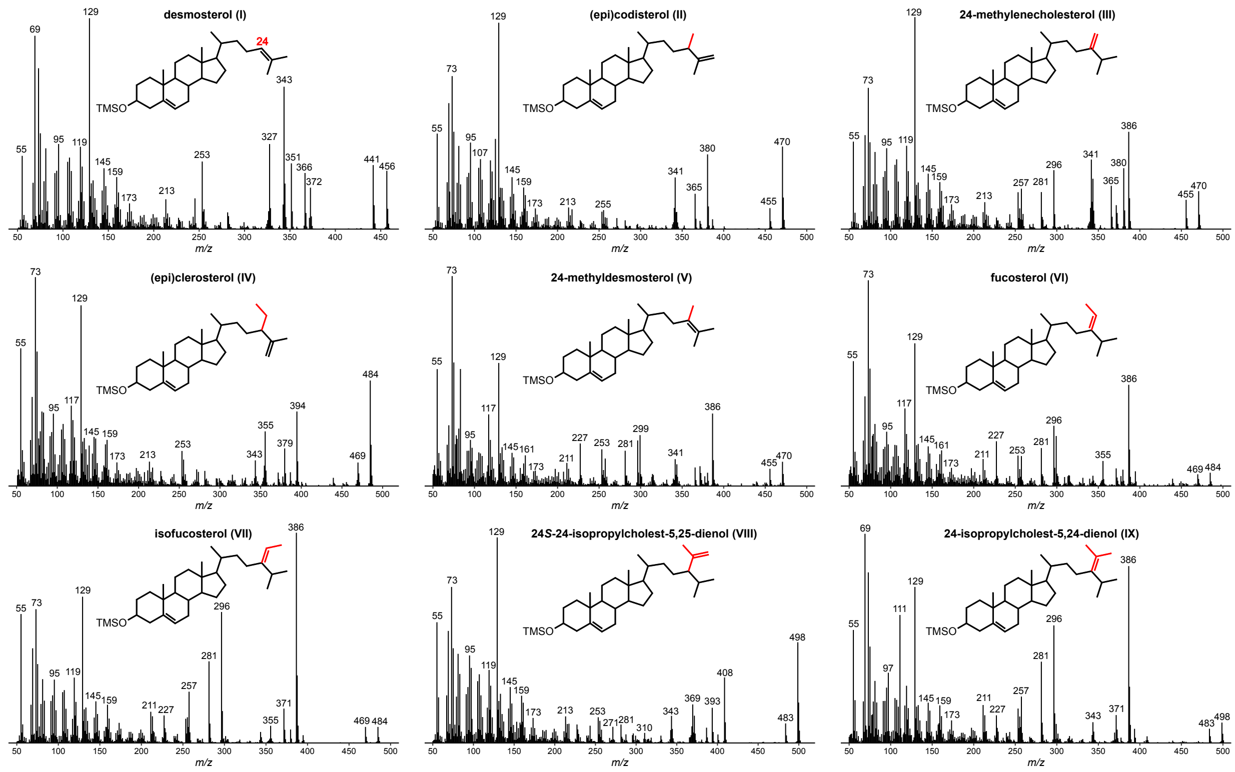


Supplementary Figure 3. **Representative mass spectra of sterols identified in the main text.** Data are from in vitro reactions performed with the "Chlamydiae sp., drinking water 1 of 2" SMT **(I, V, VI, VII, VIII, IX)** and the "*Nitrospira* sp., coral reef 1 of 2" SMT (**II**) with desmosterol as the substrate, or the "*Chondrilla nucula*" SMT with 24-methylenecholesterol as the substrate (**III, IV**). Total lipid extracts were derivatized to trimethylsilyl ethers. The raw data used to generate this figure are provided in Supplementary Data File 3. Detailed GC-MS methods can be found in the Methods section of the main text.

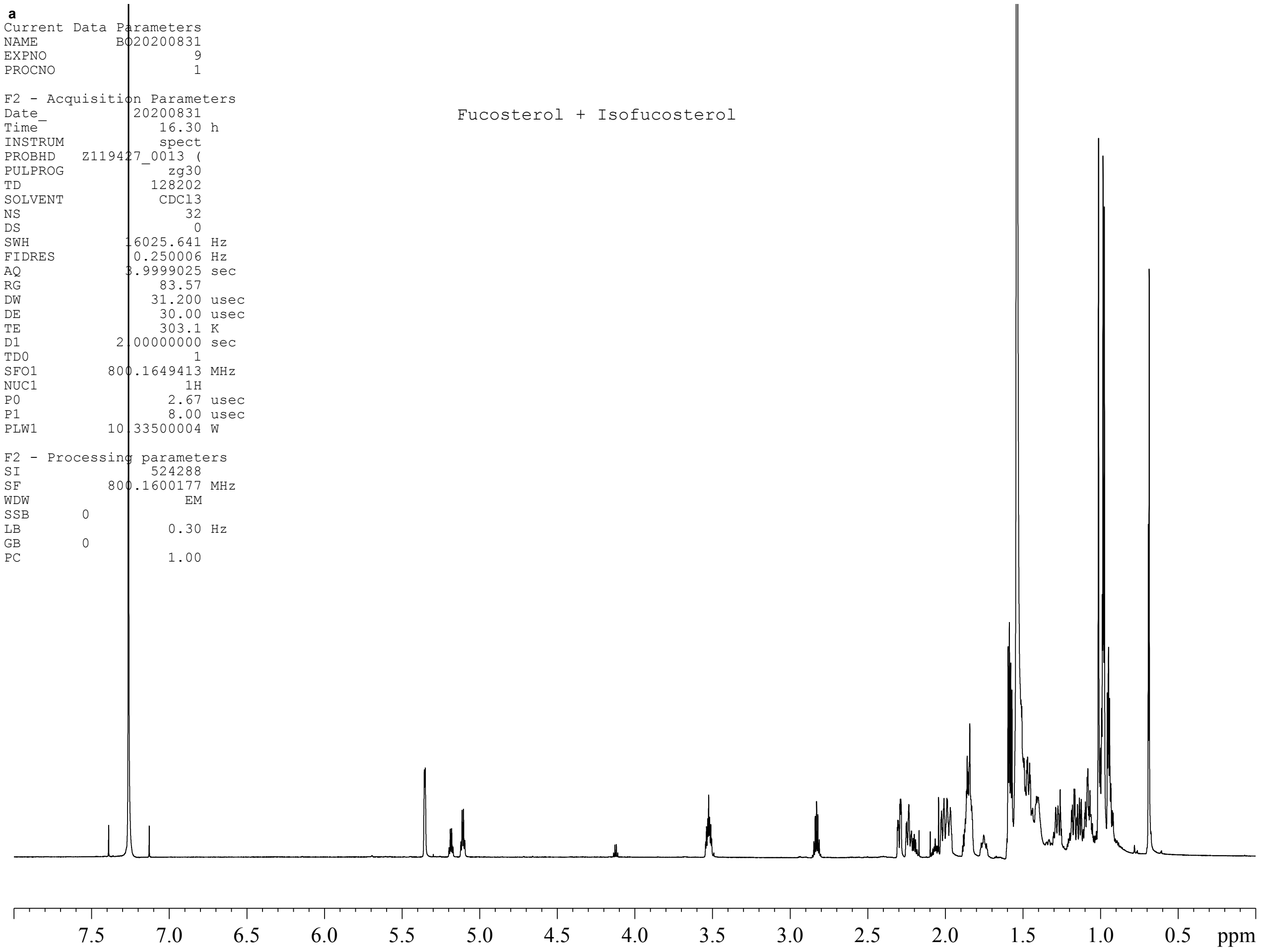


Supplementary Figure 4. **Raw 800 MHz $^{1}$H-NMR spectra of the following sterols:** **a** fucosterol plus isofucosterol, **b** clerosterol, **c** epiclerosterol, **d** 24*S*-24-isopropylcholesta-5,25-dienol, **e** 24*R*-24-isopropylcholesta-5,25-dienol, **f** [26, 27, 29, 30-$^{13}$C] 24-isopropylcholesta-5,24-dienol, and **g** [26, 27, 29, 30-$^{13}$C] 24-isopropylcholesta-5,23-dienol plus 24*S*-24-isopropylcholesta-5,25-dienol. Chemical shifts are summarized in Supplementary Table 3.

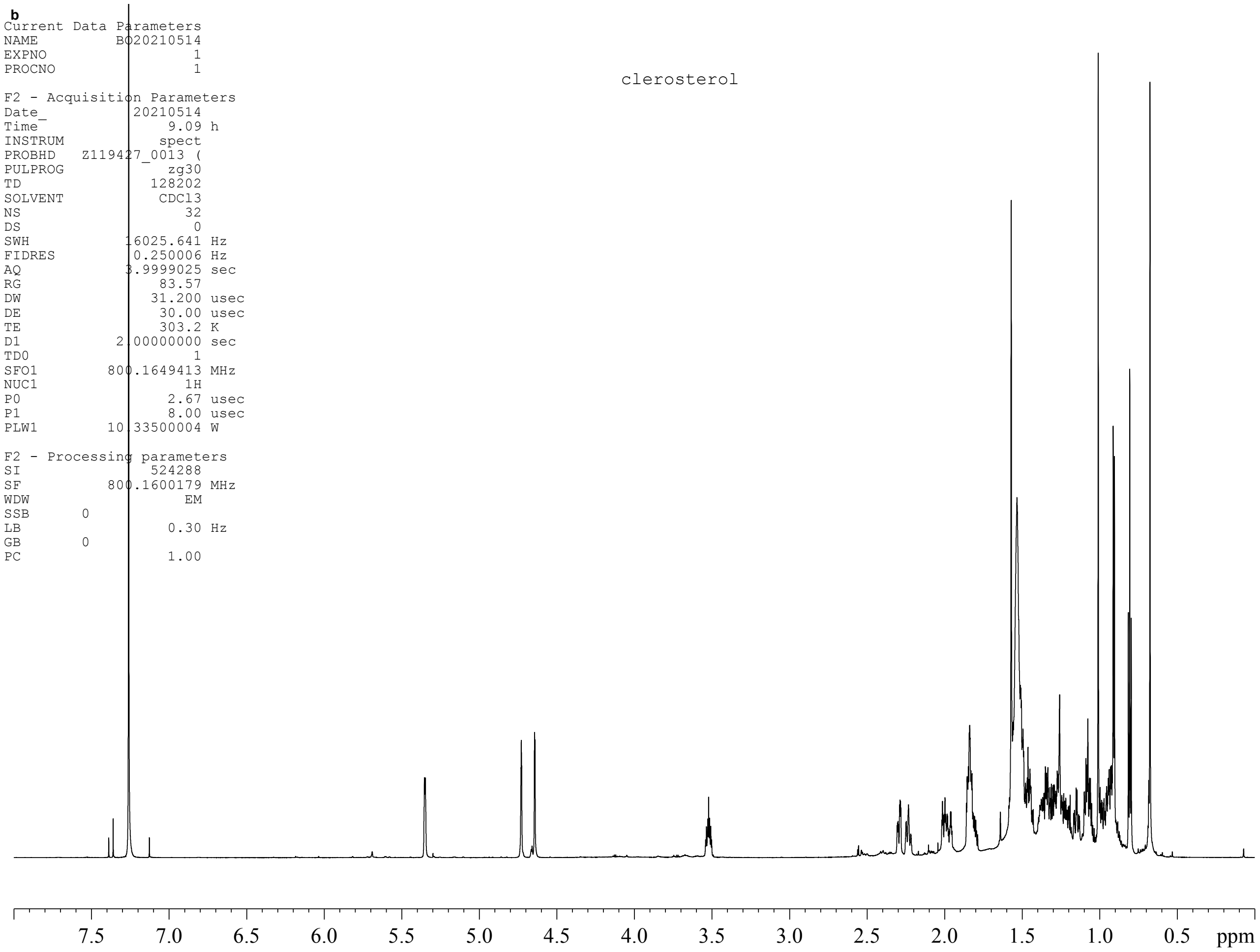
b
Current Data Parameters
NAME BO20210514
EXPNO 1
PROCNO 1
F2 - Acquisition Parameters
Date_ 20210514
Time 9.09 h
INSTRUM spect
PROBHD Z119427_0013 (
PULPROG zg30
TD 128202
SOLVENT CDCl3
NS 32
DS 0
SWH 16025.641 Hz
FIDRES 0.250006 Hz
AQ 3.9999025 sec
RG 83.57
DW 31.200 usec
DE 30.00 usec
TE 303.2 K
D1 2.00000000 sec
TD0 1
SFO1 800.1649413 MHz
NUC1 1H
P0 2.67 usec
P1 8.00 usec
PLW1 10.33500004 W
F2 - Processing parameters
SI 524288
SF 800.1600179 MHz
WDW EM
SSB 0
LB 0.30 Hz
GB 0
PC 1.00
clerosterol
7.5 7.0 6.5 6.0 5.5 5.0 4.5 4.0 3.5 3.0 2.5 2.0 1.5 1.0 0.5 ppm

c

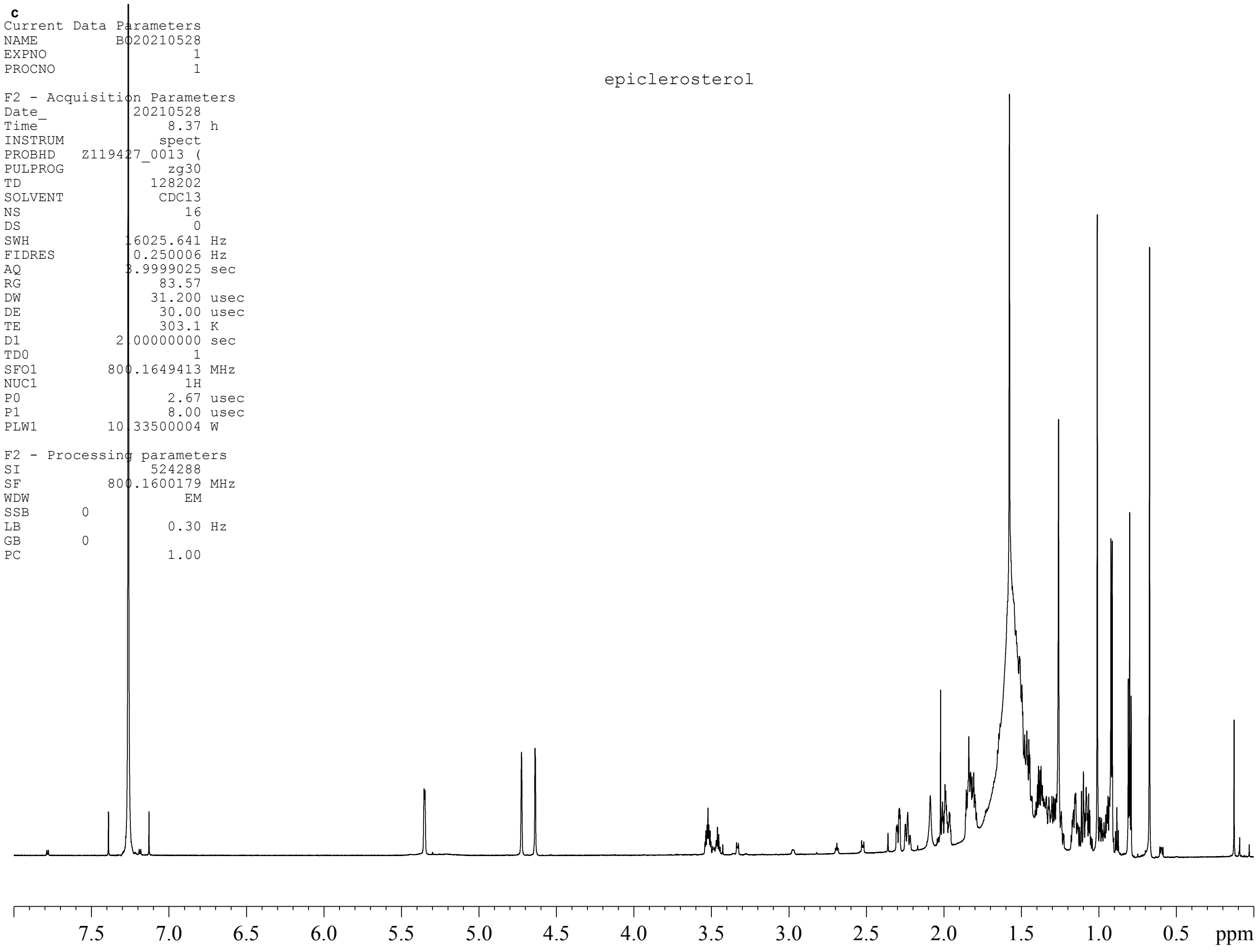
Current Data Parameters
NAME B020210528
EXPNO 1
PROCNO 1
F2 - Acquisition Parameters
Date_ 20210528
Time 8.37 h
INSTRUM spect
PROBHD Z119427_0013 (
PULPROG zg30
TD 128202
SOLVENT CDCl3
NS 16
DS 0
SWH 16025.641 Hz
FIDRES 0.250006 Hz
AQ 3.9999025 sec
RG 83.57
DW 31.200 usec
DE 30.00 usec
TE 303.1 K
D1 2.00000000 sec
TD0 1
SFO1 800.1649413 MHz
NUC1 1H
P0 2.67 usec
P1 8.00 usec
PLW1 10.33500004 W
F2 - Processing parameters
SI 524288
SF 800.1600179 MHz
WDW EM
SSB 0
LB 0.30 Hz
GB 0
PC 1.00
epiclerosterol
7.5 7.0 6.5 6.0 5.5 5.0 4.5 4.0 3.5 3.0 2.5 2.0 1.5 1.0 0.5 ppm

**d**

```
Current Data Parameters
NAME     BO20200831-800
EXPNO                 5
PROCNO                1

F2 - Acquisition Parameters
Date_          20200831
Time              14.51 h
INSTRUM           spect
PROBHD   Z119427_0013 (
PULPROG            zg30
TD               128202
SOLVENT           CDCl3
NS                   32
DS                    0
SWH           16025.641 Hz
FIDRES         0.250006 Hz
AQ            3.9999025 sec
RG                36.63
DW               31.200 usec
DE                30.00 usec
TE                303.1 K
D1           2.00000000 sec
TD0                   1
SFO1        800.1649413 MHz
NUC1                 1H
P0                 2.67 usec
P1                 8.00 usec
PLW1        10.33500004 W

F2 - Processing parameters
SI               524288
SF          800.1600179 MHz
WDW                  EM
SSB      0
LB                 0.30 Hz
GB       0
PC                 1.00
```

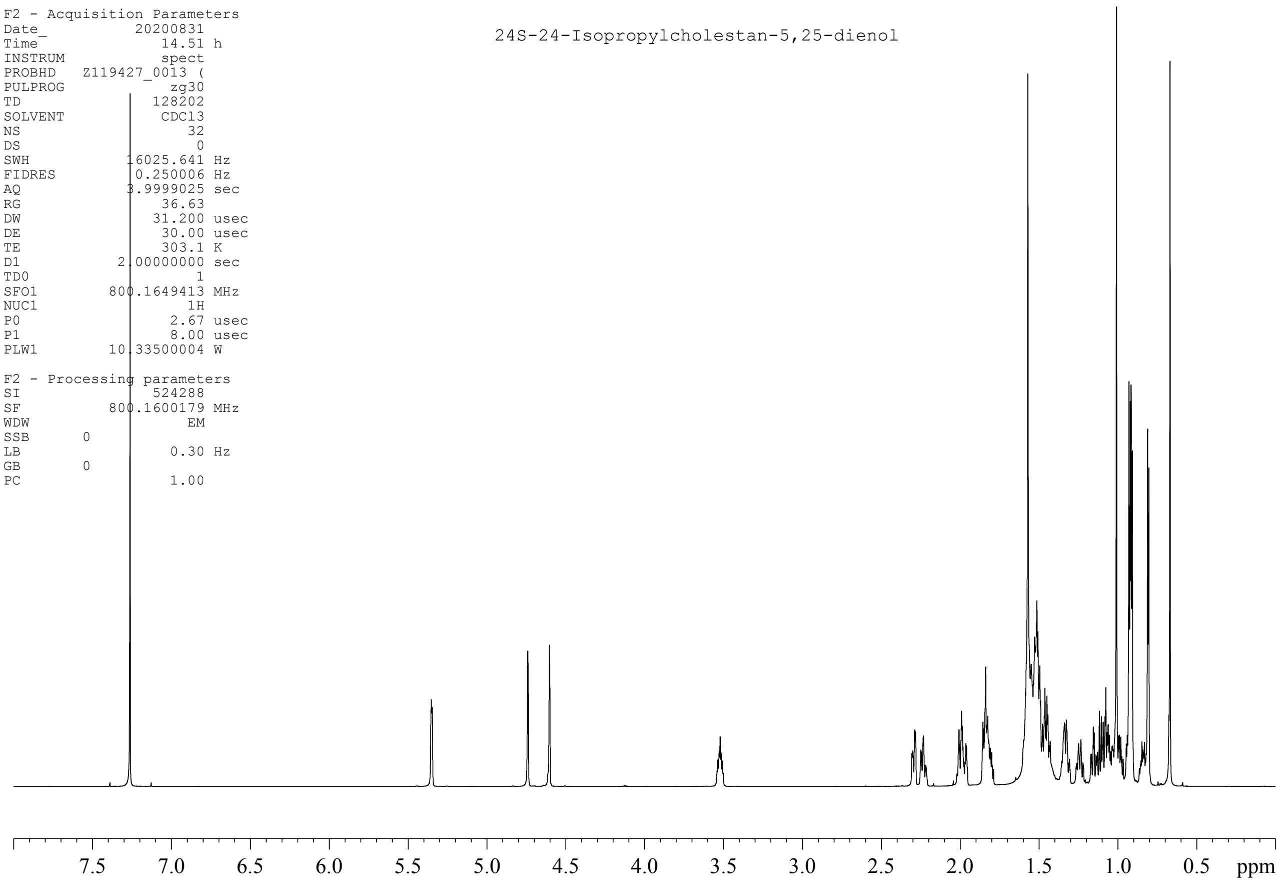

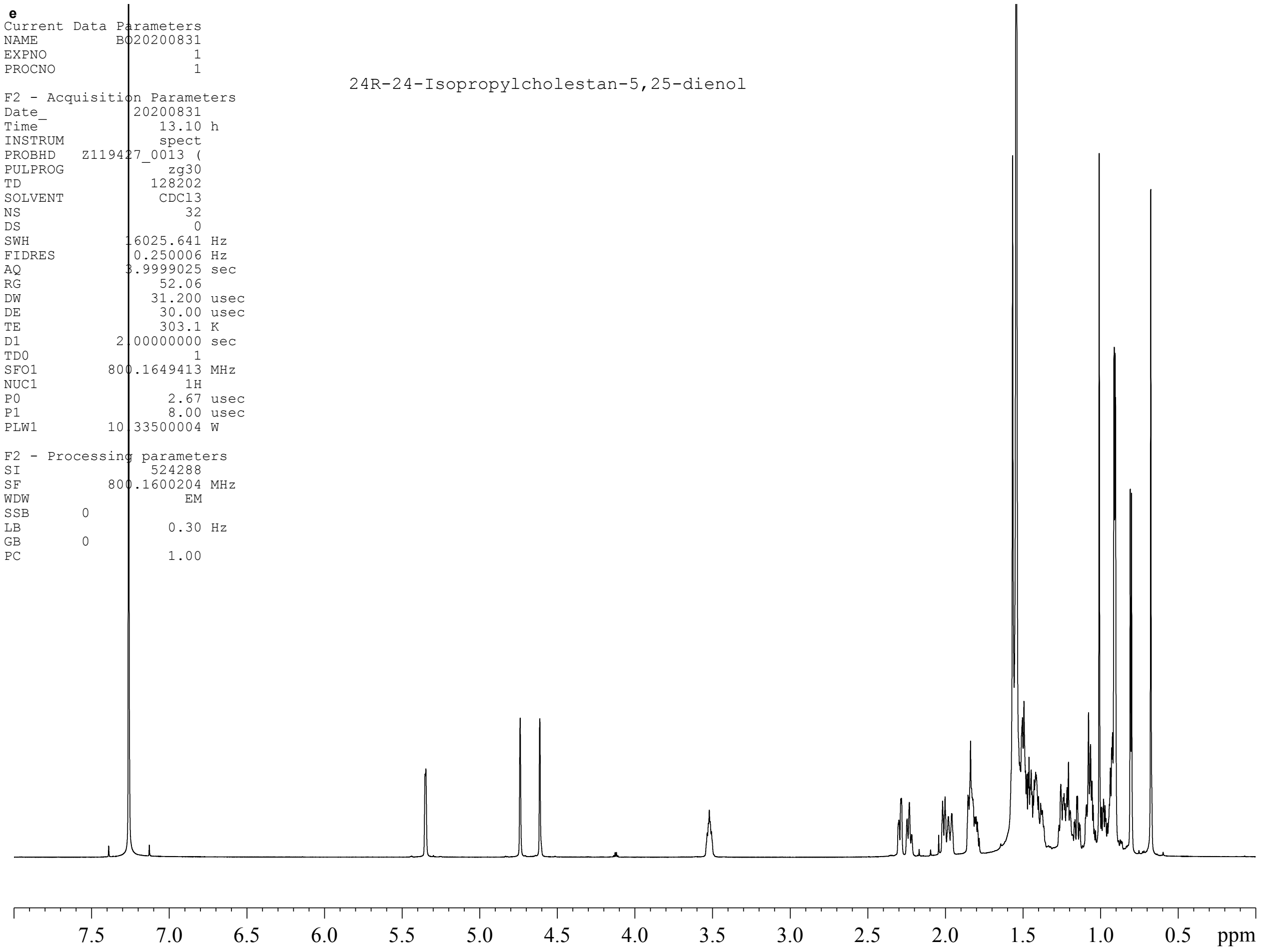
e
Current Data Parameters
NAME B020200831
EXPNO 1
PROCNO 1
F2 - Acquisition Parameters
Date_ 20200831
Time 13.10 h
INSTRUM spect
PROBHD Z119427_0013 (
PULPROG zg30
TD 128202
SOLVENT CDCl3
NS 32
DS 0
SWH 16025.641 Hz
FIDRES 0.250006 Hz
AQ 3.9999025 sec
RG 52.06
DW 31.200 usec
DE 30.00 usec
TE 303.1 K
D1 2.00000000 sec
TD0 1
SFO1 800.1649413 MHz
NUC1 1H
P0 2.67 usec
P1 8.00 usec
PLW1 10.33500004 W
F2 - Processing parameters
SI 524288
SF 800.1600204 MHz
WDW EM
SSB 0
LB 0.30 Hz
GB 0
PC 1.00
24R-24-Isopropylcholestan-5,25-dienol
7.5 7.0 6.5 6.0 5.5 5.0 4.5 4.0 3.5 3.0 2.5 2.0 1.5 1.0 0.5 ppm

**f**

```
Current Data Parameters
NAME         B020210403
EXPNO                12
PROCNO                1

F2 - Acquisition Parameters
Date_          20210403
Time              14.57 h
INSTRUM           spect
PROBHD   Z119427_0013 (
PULPROG            zg30
TD               128202
SOLVENT           CDCl3
NS                   32
DS                    0
SWH           16025.641 Hz
FIDRES         0.250006 Hz
AQ            3.9999025 sec
RG                83.57
DW               31.200 usec
DE                30.00 usec
TE                303.2 K
D1           2.00000000 sec
TD0                   1
SFO1        800.1649413 MHz
NUC1                 1H
P0                 2.67 usec
P1                 8.00 usec
PLW1        10.33500004 W

F2 - Processing parameters
SI               524288
SF          800.1600180 MHz
WDW                  EM
SSB      0
LB                 0.30 Hz
GB       0
PC                 1.00
```

26,27,29,30 - [13C] 24-Isopropylcholestan-5,24-dienol

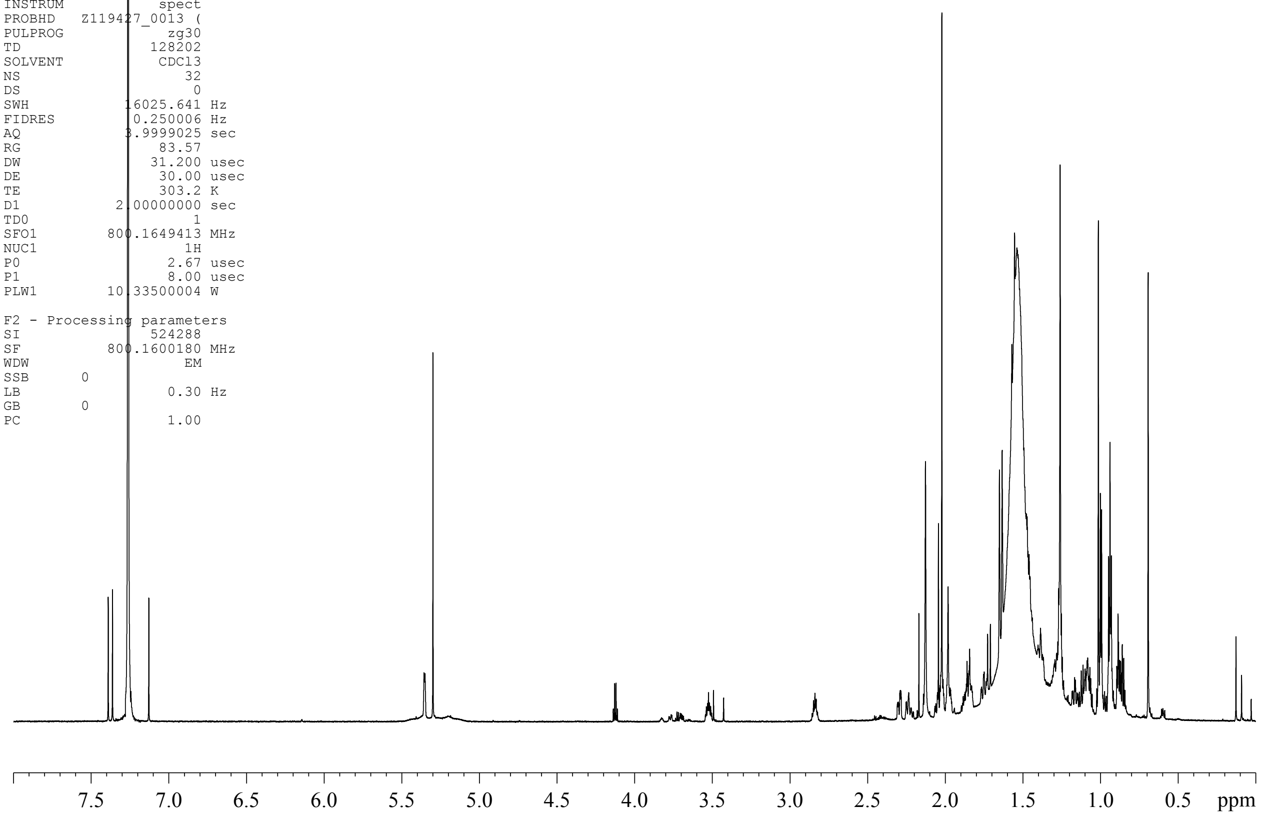

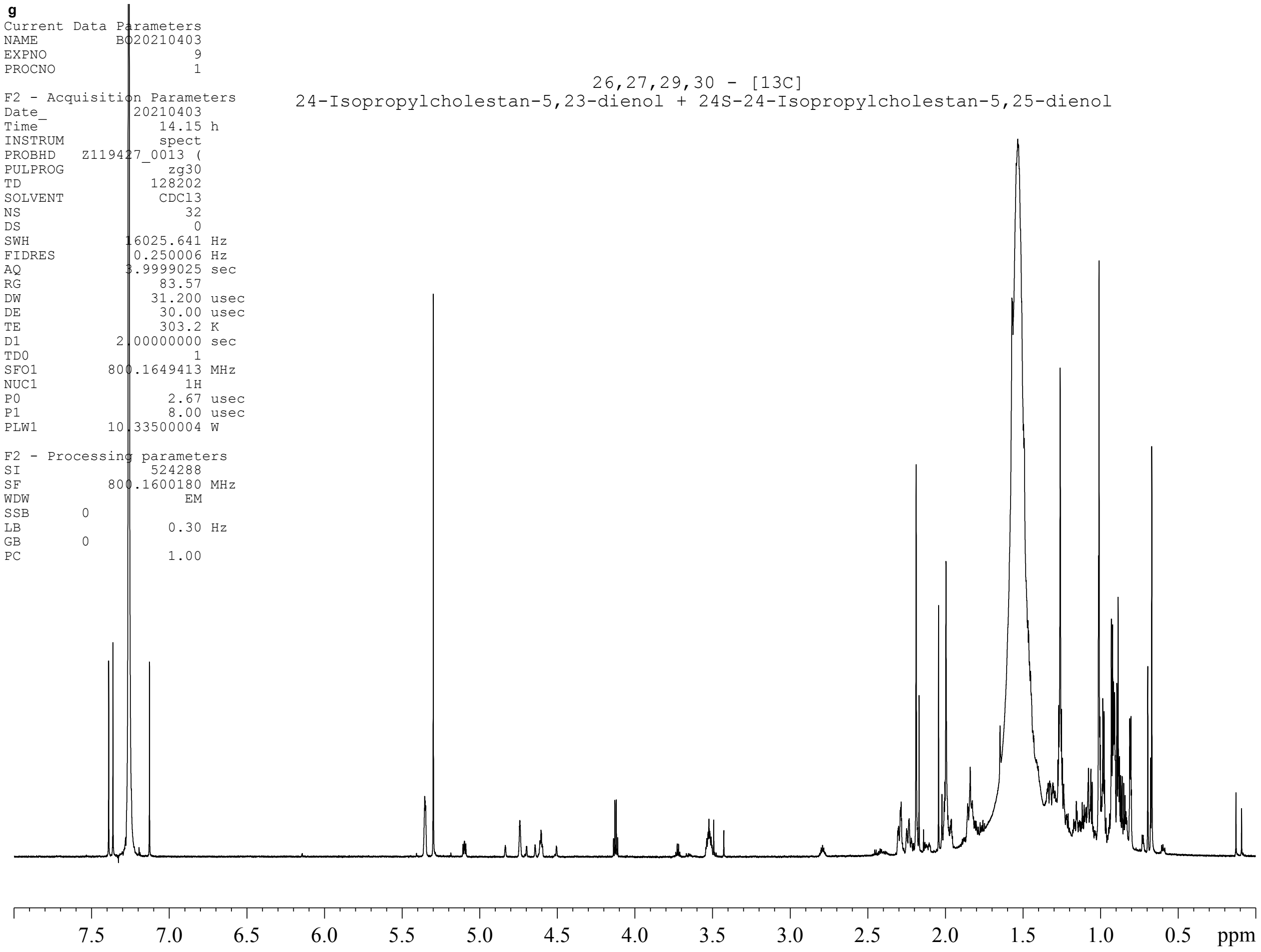
g
Current Data Parameters
NAME B020210403
EXPNO 9
PROCNO 1
F2 - Acquisition Parameters
Date_ 20210403
Time 14.15 h
INSTRUM spect
PROBHD Z119427_0013 (
PULPROG zg30
TD 128202
SOLVENT CDCl3
NS 32
DS 0
SWH 16025.641 Hz
FIDRES 0.250006 Hz
AQ 3.9999025 sec
RG 83.57
DW 31.200 usec
DE 30.00 usec
TE 303.2 K
D1 2.00000000 sec
TD0 1
SFO1 800.1649413 MHz
NUC1 1H
P0 2.67 usec
P1 8.00 usec
PLW1 10.33500004 W
F2 - Processing parameters
SI 524288
SF 800.1600180 MHz
WDW EM
SSB 0
LB 0.30 Hz
GB 0
PC 1.00
26,27,29,30 - [13C]
24-Isopropylcholestan-5,23-dienol + 24S-24-Isopropylcholestan-5,25-dienol
7.5
7.0
6.5
6.0
5.5
5.0
4.5
4.0
3.5
3.0
2.5
2.0
1.5
1.0
0.5
ppm

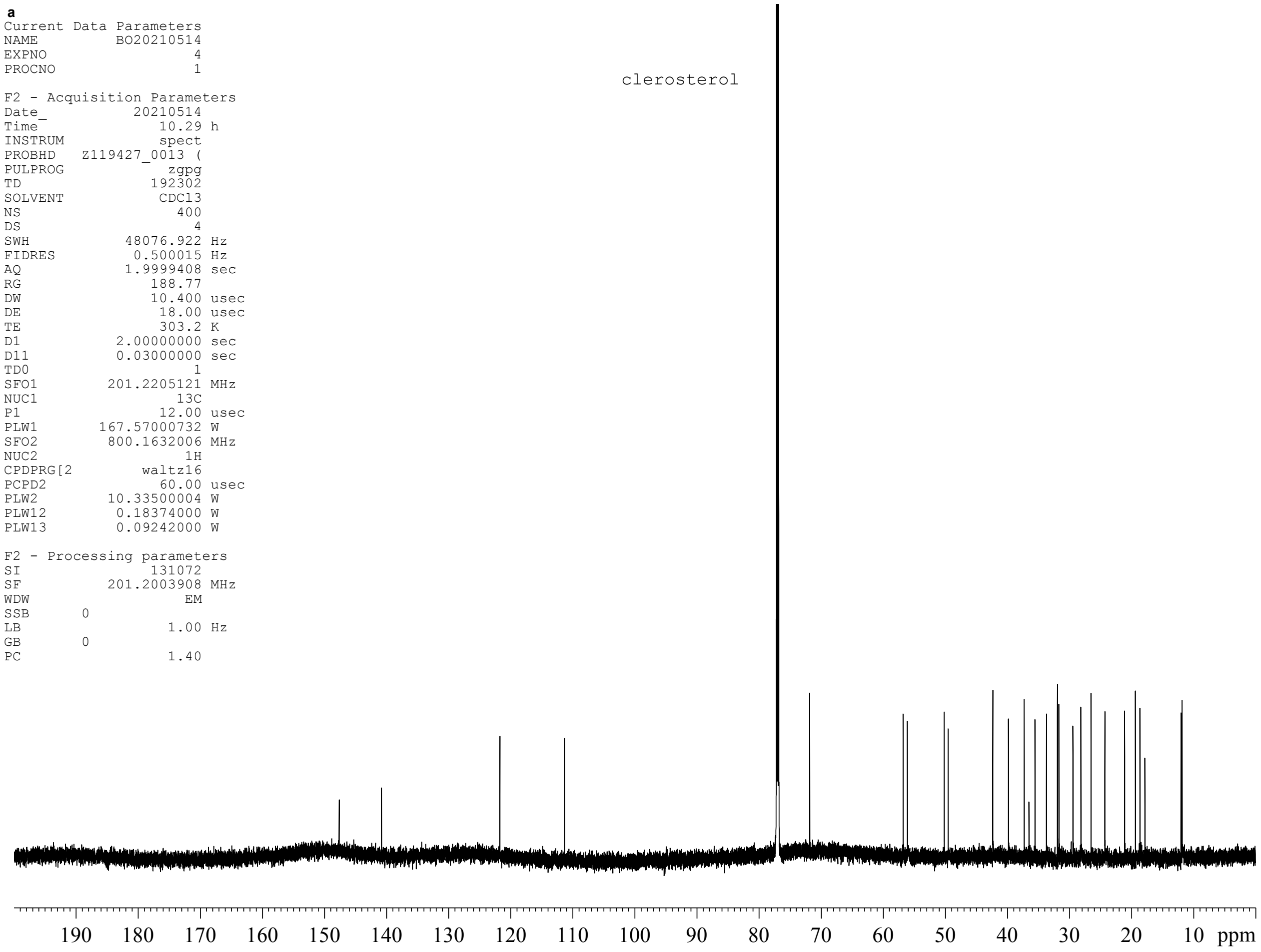


Supplementary Figure 5. **Raw 201 MHz $^{13}$C-NMR spectra of the following sterols: a** clerosterol, **b** epiclerosterol, **c** 24*S*-24-isopropylcholesta-5,25-dienol **d** 24*R*-24-isopropylcholesta-5,25-dienol, **e** [26, 27, 29, 30-$^{13}$C] 24-isopropylcholesta-5,24-dienol, and **f** [26, 27, 29, 30-$^{13}$C] 24-isopropylcholesta-5,23-dienol plus 24*S*-24-isopropylcholesta-5,25-dienol. Chemical shifts are summarized in Supplementary Table 3.

**b**

```
Current Data Parameters
NAME          BO20210528
EXPNO                  2
PROCNO                 1

F2 - Acquisition Parameters
Date_           20210528
Time                9.35 h
INSTRUM            spect
PROBHD   Z119427_0013 (
PULPROG             zgpg
TD                192302
SOLVENT            CDCl3
NS                   800
DS                     4
SWH            48076.922 Hz
FIDRES          0.500015 Hz
AQ             1.9999408 sec
RG                188.77
DW                10.400 usec
DE                 18.00 usec
TE                 303.1 K
D1            2.00000000 sec
D11           0.03000000 sec
TD0                    1
SFO1         201.2205121 MHz
NUC1                 13C
P1                 12.00 usec
PLW1        167.57000732 W
SFO2         800.1632006 MHz
NUC2                  1H
CPDPRG[2         waltz16
PCPD2              60.00 usec
PLW2         10.33500004 W
PLW12         0.18374000 W
PLW13         0.09242000 W

F2 - Processing parameters
SI                131072
SF           201.2003908 MHz
WDW                   EM
SSB      0
LB                  1.00 Hz
GB       0
PC                  1.40
```

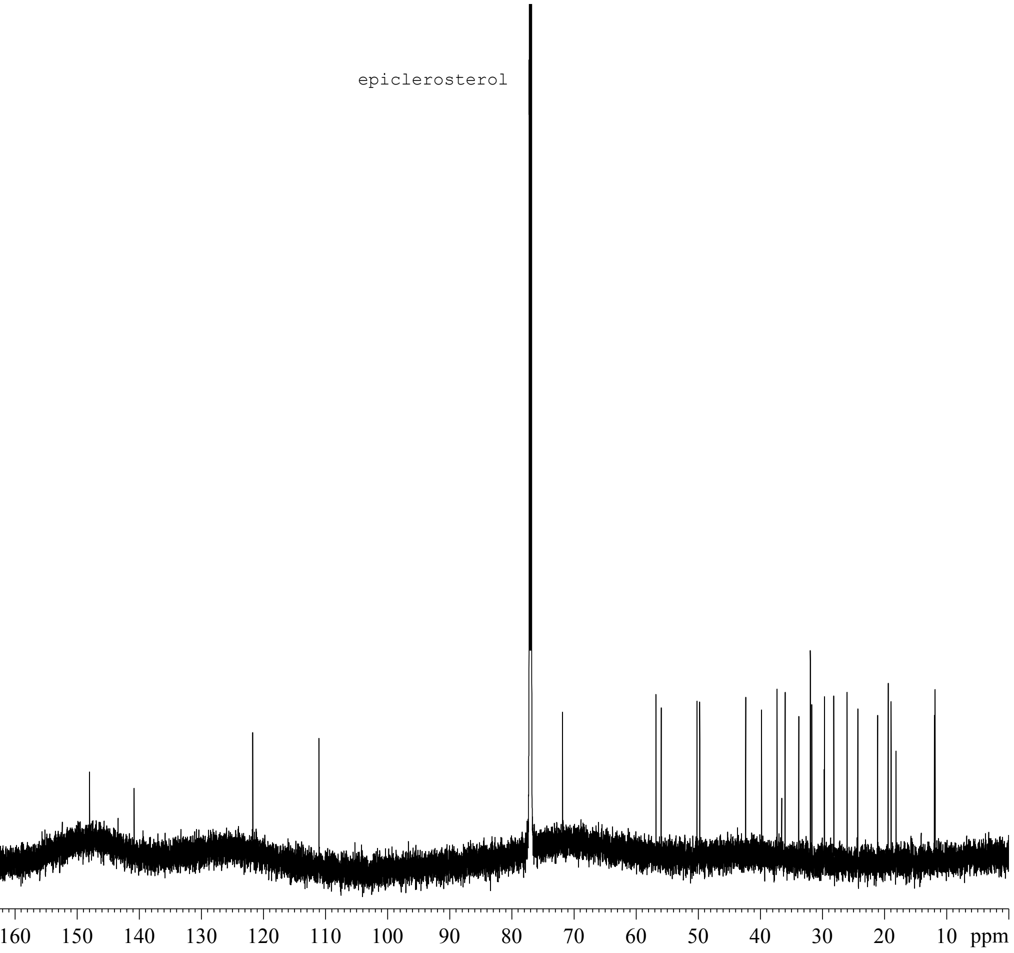

**c**

```
Current Data Parameters
NAME         BO20200831
EXPNO                 6
PROCNO                1

F2 - Acquisition Parameters
Date_          20200831
Time              15.17 h
INSTRUM           spect
PROBHD   Z119427_0013 (
PULPROG            zgpg
TD               192302
SOLVENT           CDCl3
NS                  256
DS                    4
SWH           48076.922 Hz
FIDRES         0.500015 Hz
AQ            1.9999408 sec
RG               188.77
DW               10.400 usec
DE                18.00 usec
TE                300.0 K
D1           2.00000000 sec
D11          0.03000000 sec
TD0                   1
SFO1        201.2205121 MHz
NUC1                13C
P1                12.00 usec
PLW1       167.57000732 W
SFO2        800.1632006 MHz
NUC2                 1H
CPDPRG[2        waltz16
PCPD2             60.00 usec
PLW2        10.33500004 W
PLW12        0.18374000 W
PLW13        0.09242000 W

F2 - Processing parameters
SI               131072
SF          201.2003945 MHz
WDW                  EM
SSB      0
LB                 1.00 Hz
GB       0
PC                 1.40
```

24S-24-Isopropylcholestan-5,25-dienol

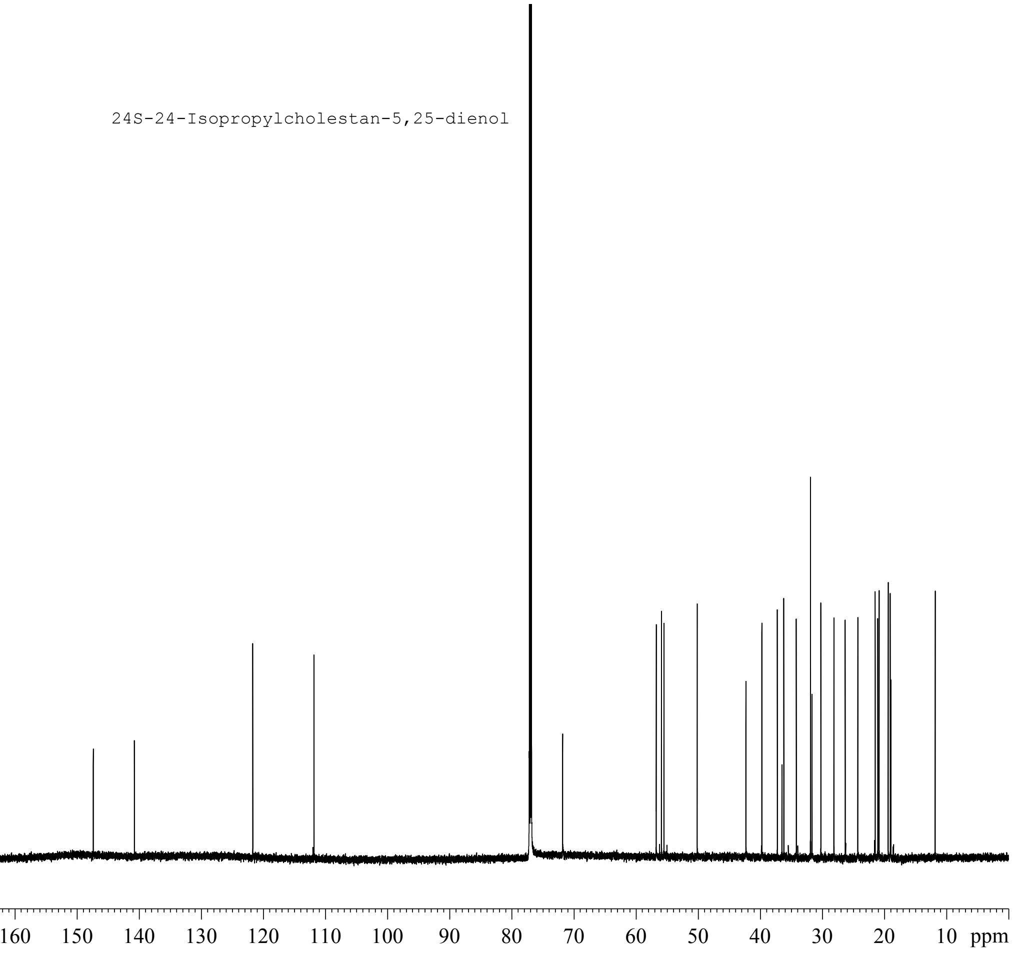

d

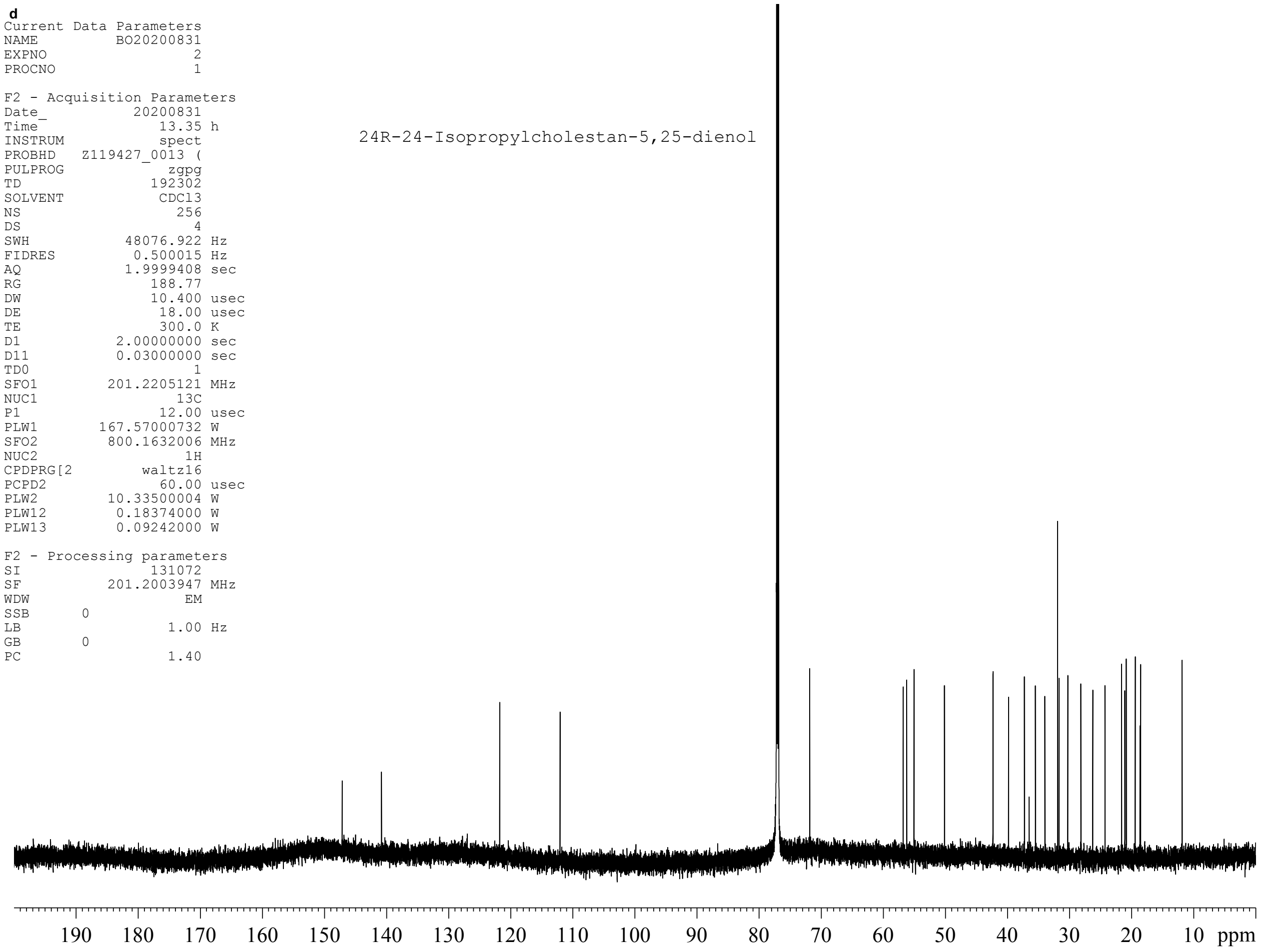
Current Data Parameters
NAME BO20200831
EXPNO 2
PROCNO 1
F2 - Acquisition Parameters
Date_ 20200831
Time 13.35 h
INSTRUM spect
PROBHD Z119427_0013 (
PULPROG zgpg
TD 192302
SOLVENT CDCl3
NS 256
DS 4
SWH 48076.922 Hz
FIDRES 0.500015 Hz
AQ 1.9999408 sec
RG 188.77
DW 10.400 usec
DE 18.00 usec
TE 300.0 K
D1 2.00000000 sec
D11 0.03000000 sec
TD0 1
SFO1 201.2205121 MHz
NUC1 13C
P1 12.00 usec
PLW1 167.57000732 W
SFO2 800.1632006 MHz
NUC2 1H
CPDPRG[2 waltz16
PCPD2 60.00 usec
PLW2 10.33500004 W
PLW12 0.18374000 W
PLW13 0.09242000 W
F2 - Processing parameters
SI 131072
SF 201.2003947 MHz
WDW EM
SSB 0
LB 1.00 Hz
GB 0
PC 1.40
24R-24-Isopropylcholestan-5,25-dienol
190 180 170 160 150 140 130 120 110 100 90 80 70 60 50 40 30 20 10 ppm

**e**

```
Current Data Parameters
NAME         BO20210403
EXPNO                13
PROCNO                1

F2 - Acquisition Parameters
Date_          20210403
Time              15.07 h
INSTRUM           spect
PROBHD   Z119427_0013 (
PULPROG            zgpg
TD               192302
SOLVENT           CDCl3
NS                  128
DS                    4
SWH           48076.922 Hz
FIDRES         0.500015 Hz
AQ            1.9999408 sec
RG               188.77
DW               10.400 usec
DE                18.00 usec
TE                303.2 K
D1           2.00000000 sec
D11          0.03000000 sec
TD0                   1
SFO1        201.2205121 MHz
NUC1                13C
P1                12.00 usec
PLW1       167.57000732 W
SFO2        800.1632006 MHz
NUC2                 1H
CPDPRG[2        waltz16
PCPD2             60.00 usec
PLW2        10.33500004 W
PLW12        0.18374000 W
PLW13        0.09242000 W

F2 - Processing parameters
SI               131072
SF          201.2003918 MHz
WDW                  EM
SSB      0
LB                 1.00 Hz
GB       0
PC                 1.40
```

26,27,29,30 - [13C]
24-Isopropylcholestan-5,24-dienol

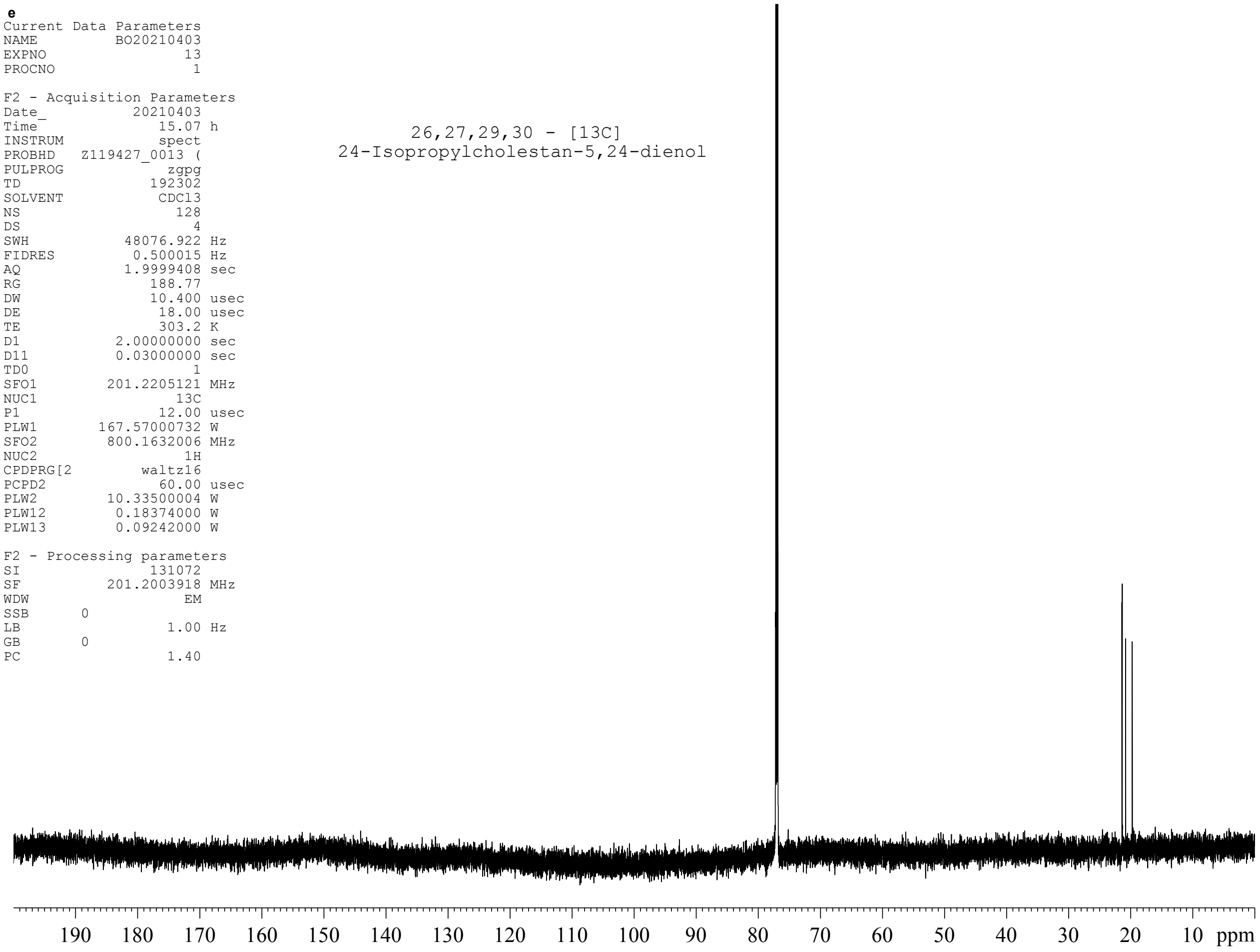

**f**

```
Current Data Parameters
NAME         BO20210403
EXPNO                10
PROCNO                1

F2 - Acquisition Parameters
Date_          20210403
Time              14.24 h
INSTRUM           spect
PROBHD   Z119427_0013 (
PULPROG            zgpg
TD               192302
SOLVENT           CDCl3
NS                  128
DS                    4
SWH           48076.922 Hz
FIDRES         0.500015 Hz
AQ            1.9999408 sec
RG               188.77
DW               10.400 usec
DE                18.00 usec
TE                303.1 K
D1           2.00000000 sec
D11          0.03000000 sec
TD0                   1
SFO1        201.2205121 MHz
NUC1                13C
P1                12.00 usec
PLW1       167.57000732 W
SFO2        800.1632006 MHz
NUC2                 1H
CPDPRG[2        waltz16
PCPD2             60.00 usec
PLW2        10.33500004 W
PLW12        0.18374000 W
PLW13        0.09242000 W

F2 - Processing parameters
SI               131072
SF          201.2003916 MHz
WDW                  EM
SSB      0
LB                 1.00 Hz
GB       0
PC                 1.40
```

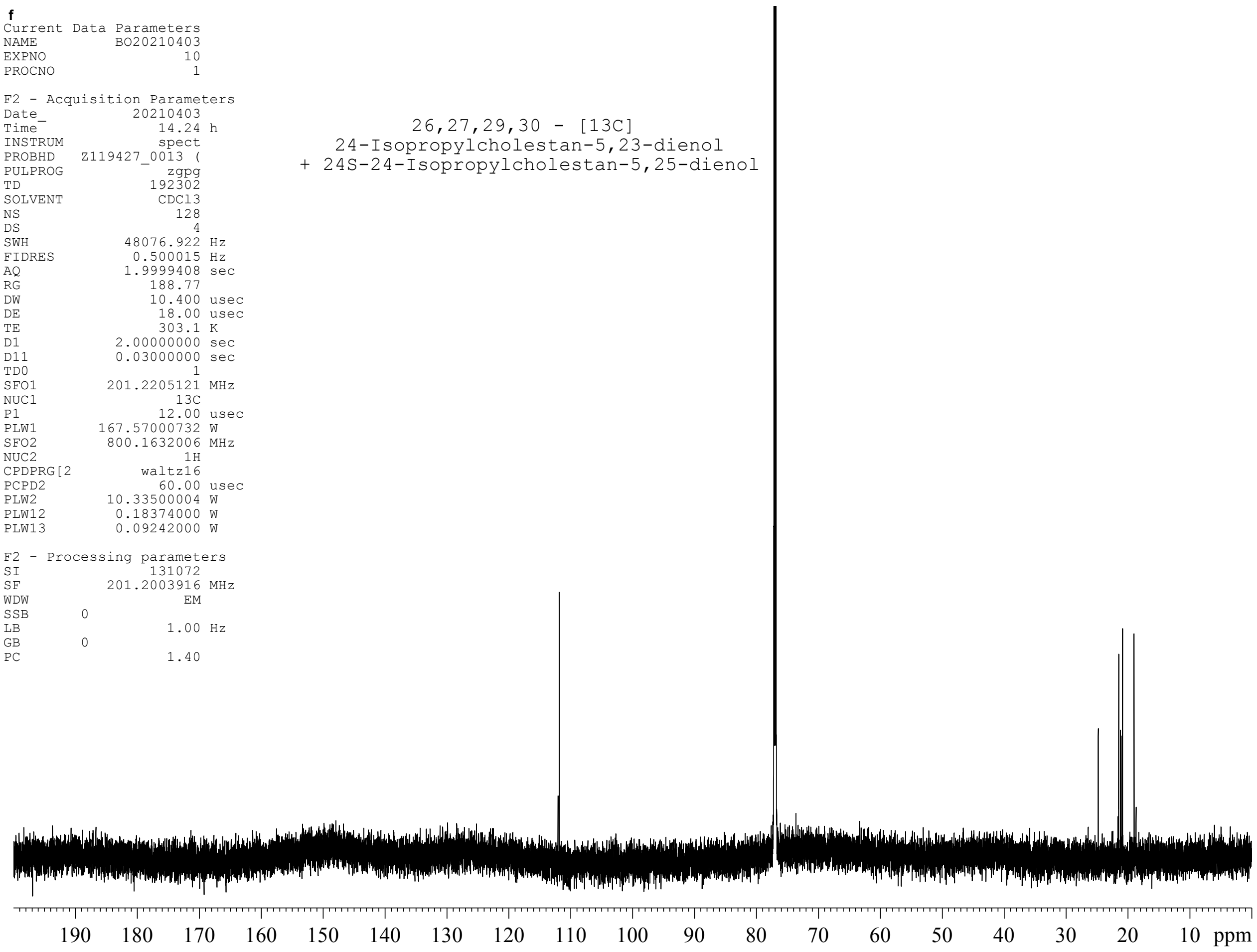

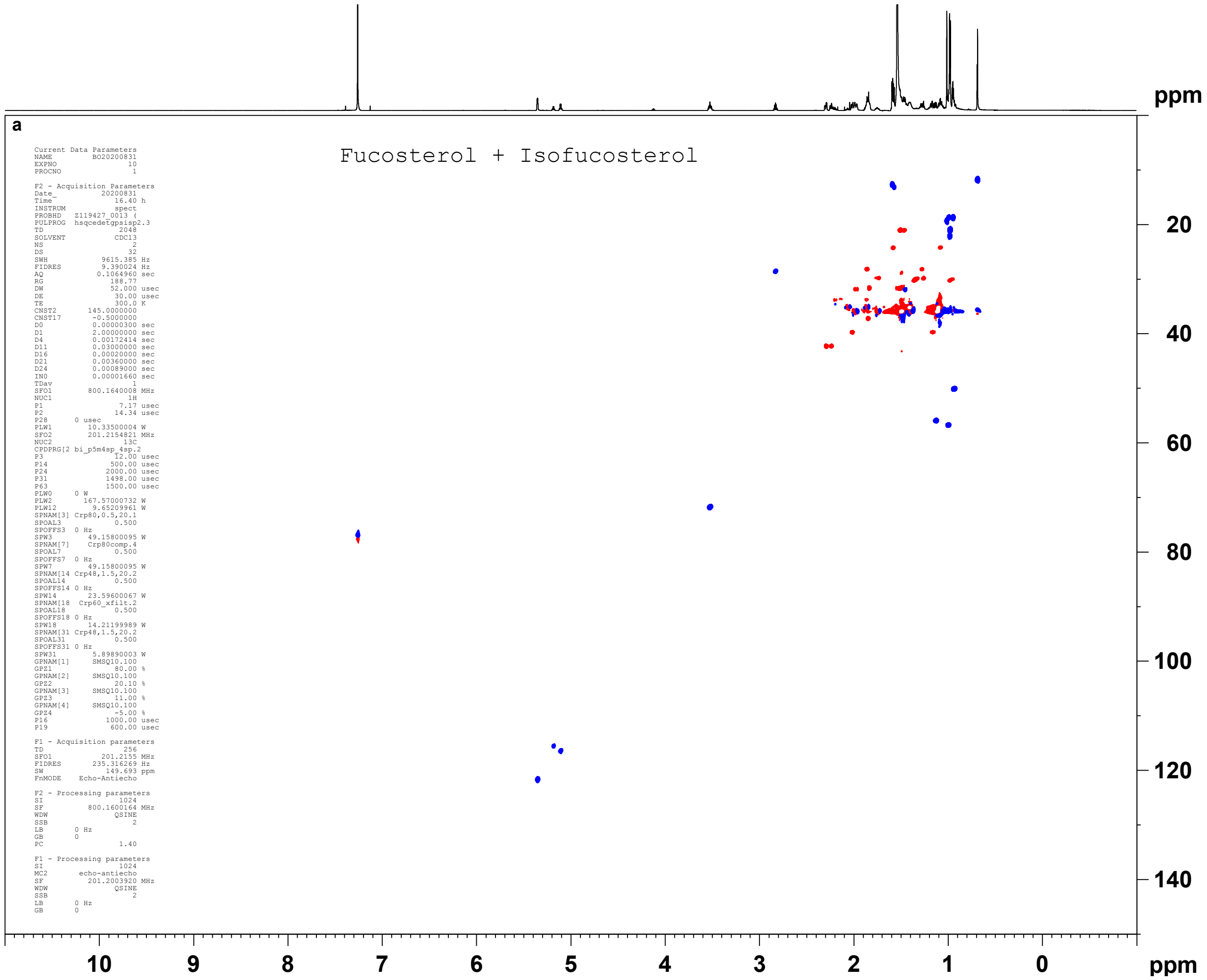


Supplementary Figure 6. **Raw 2D NMR (HSQC) spectra of the following sterols: a** fucosterol plus isofucosterol, **b** clerosterol, **c** epiclerosterol, **d** 24*S*-24-isopropylcholesta-5,25-dienol, **e** 24*R*-24-isopropylcholesta-5,25-dienol, **f** [26, 27, 29, 30-$^{13}$C] 24-isopropylcholesta-5,24-dienol, and **g** [26, 27, 29, 30-$^{13}$C] 24-isopropylcholest-5,23-dienol plus 24*S*-24-isopropylcholesta-5,25-dienol. Chemical shifts are summarized in Supplementary Table 3.

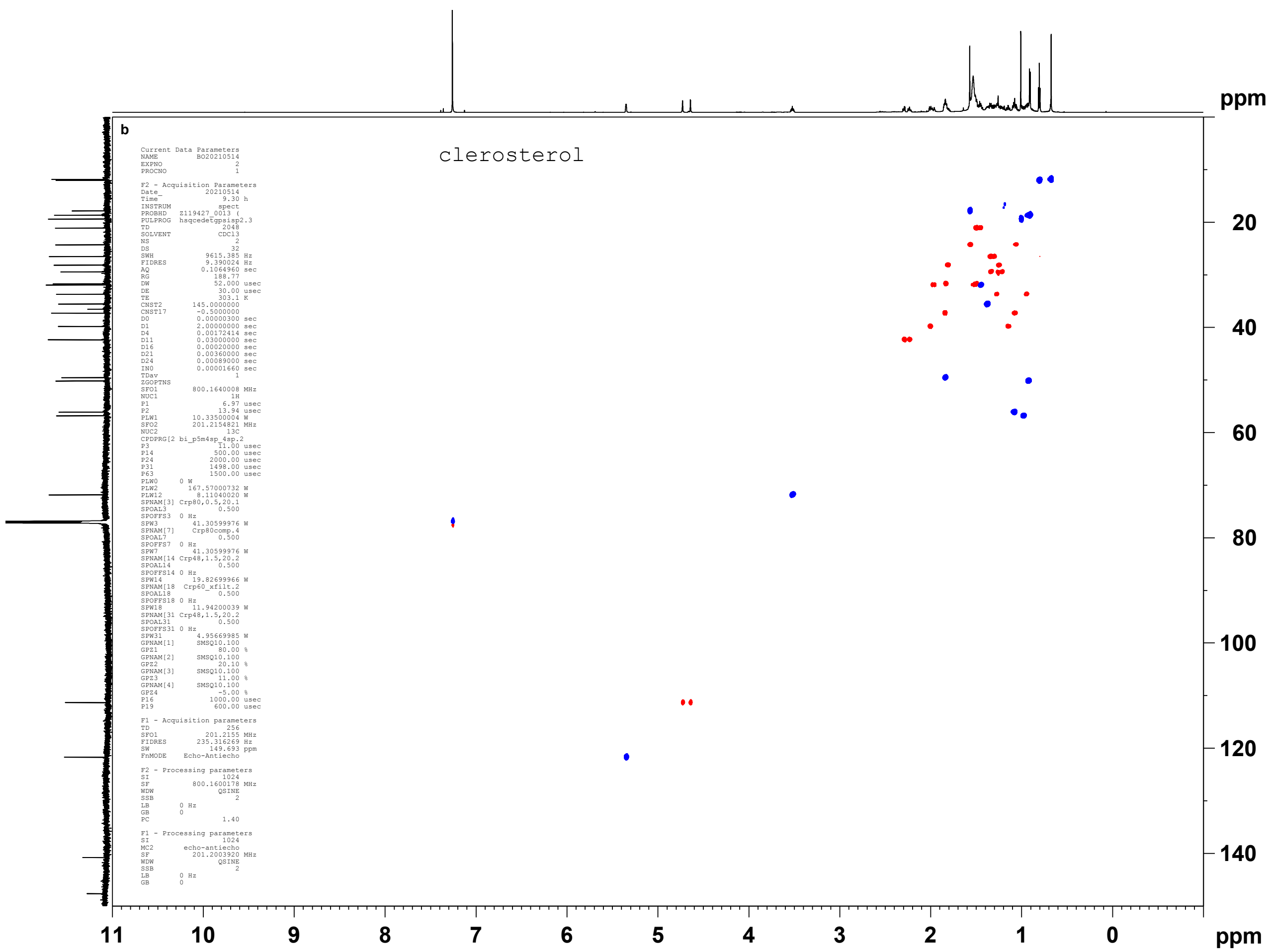
b
clerosterol
Current Data Parameters
NAME BO20210514
EXPNO 2
PROCNO 1
F2 - Acquisition Parameters
Date_ 20210514
Time 9.30 h
INSTRUM spect
PROBHD Z119427_0013 (
PULPROG hsqcedetgpsisp2.3
TD 2048
SOLVENT CDCl3
NS 2
DS 32
SWH 9615.385 Hz
FIDRES 9.390024 Hz
AQ 0.1064960 sec
RG 188.77
DW 52.000 usec
DE 30.00 usec
TE 303.1 K
CNST2 145.0000000
CNST17 -0.5000000
D0 0.00000300 sec
D1 2.00000000 sec
D4 0.00172414 sec
D11 0.03000000 sec
D16 0.00020000 sec
D21 0.00360000 sec
D24 0.00089000 sec
IN0 0.00001660 sec
TDav 1
ZGOPTNS
SFO1 800.1640008 MHz
NUC1 1H
P1 6.97 usec
P2 13.94 usec
PLW1 10.33500004 W
SFO2 201.2154821 MHz
NUC2 13C
CPDPRG[2 bi_p5m4sp_4sp.2
P3 11.00 usec
P14 500.00 usec
P24 2000.00 usec
P31 1498.00 usec
P63 1500.00 usec
PLW0 0 W
PLW2 167.57000732 W
PLW12 8.11040020 W
SPNAM[3] Crp80,0.5,20.1
SPOAL3 0.500
SPOFFS3 0 Hz
SPW3 41.30599976 W
SPNAM[7] Crp80comp.4
SPOAL7 0.500
SPOFFS7 0 Hz
SPW7 41.30599976 W
SPNAM[14 Crp48,1.5,20.2
SPOAL14 0.500
SPOFFS14 0 Hz
SPW14 19.82699966 W
SPNAM[18 Crp60_xfilt.2
SPOAL18 0.500
SPOFFS18 0 Hz
SPW18 11.94200039 W
SPNAM[31 Crp48,1.5,20.2
SPOAL31 0.500
SPOFFS31 0 Hz
SPW31 4.95669985 W
GPNAM[1] SMSQ10.100
GPZ1 80.00 %
GPNAM[2] SMSQ10.100
GPZ2 20.10 %
GPNAM[3] SMSQ10.100
GPZ3 11.00 %
GPNAM[4] SMSQ10.100
GPZ4 -5.00 %
P16 1000.00 usec
P19 600.00 usec
F1 - Acquisition parameters
TD 256
SFO1 201.2155 MHz
FIDRES 235.316269 Hz
SW 149.693 ppm
FnMODE Echo-Antiecho
F2 - Processing parameters
SI 1024
SF 800.1600178 MHz
WDW QSINE
SSB 2
LB 0 Hz
GB 0
PC 1.40
F1 - Processing parameters
SI 1024
MC2 echo-antiecho
SF 201.2003920 MHz
WDW QSINE
SSB 2
LB 0 Hz
GB 0
ppm
20
40
60
80
100
120
140
11
10
9
8
7
6
5
4
3
2
1
0
ppm

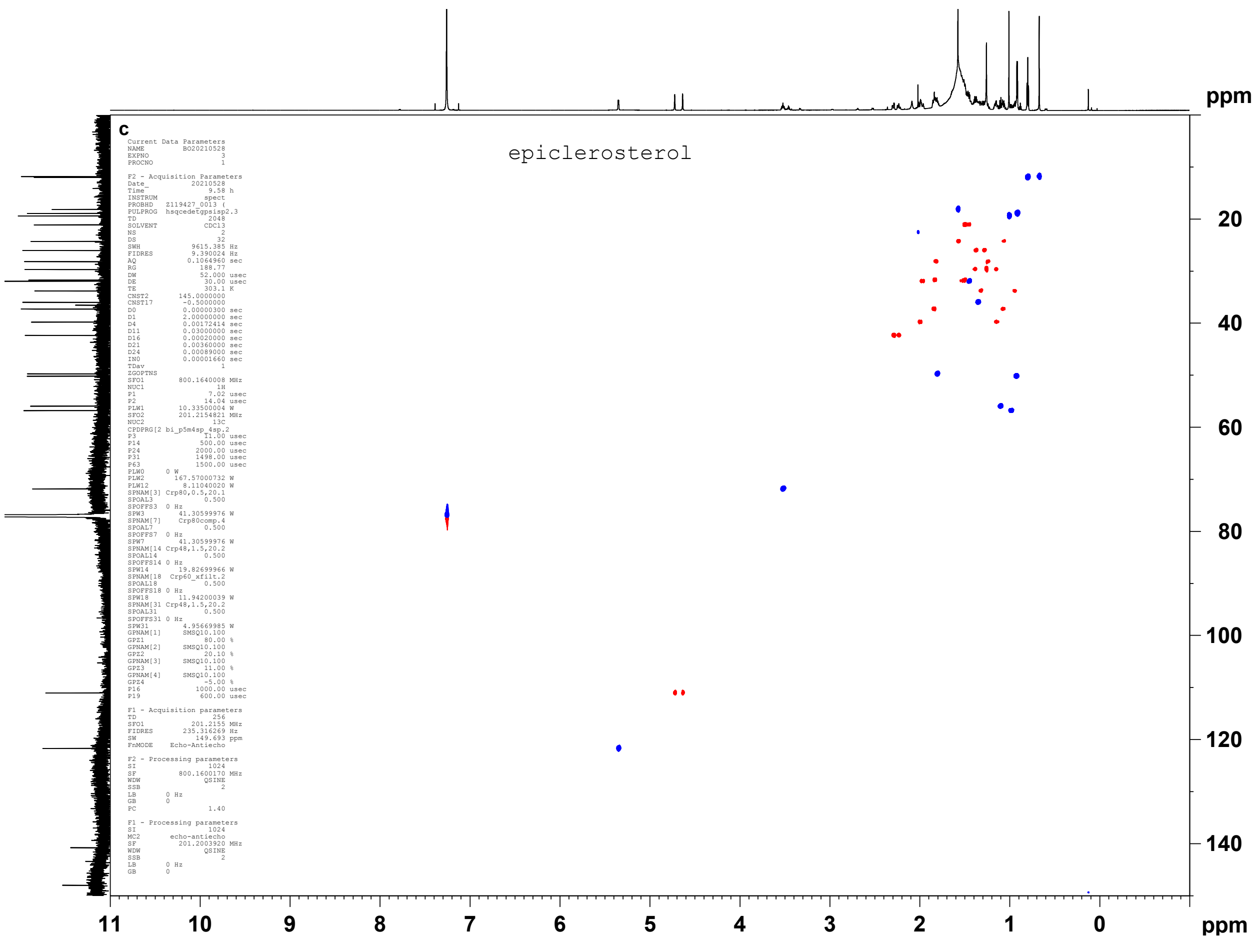
c
epiclerosterol
ppm
20
40
60
80
100
120
140
11 10 9 8 7 6 5 4 3 2 1 0
ppm

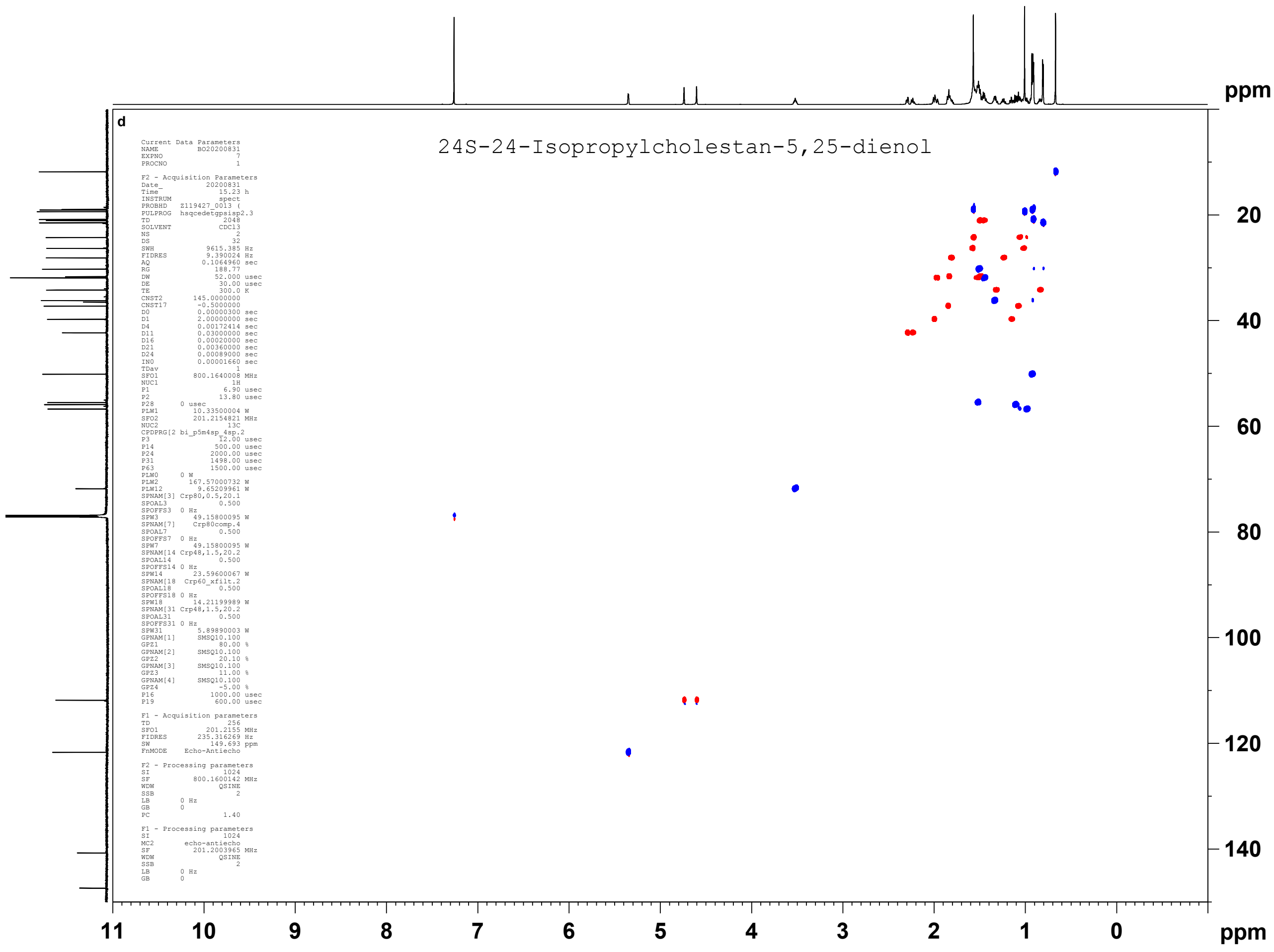
d
24S-24-Isopropylcholestan-5,25-dienol
Current Data Parameters
NAME BO20200831
EXPNO 7
PROCNO 1
F2 - Acquisition Parameters
Date_ 20200831
Time 15.23 h
INSTRUM spect
PROBHD Z119427_0013 (
PULPROG hsqcedetgpsisp2.3
TD 2048
SOLVENT CDCl3
NS 2
DS 32
SWH 9615.385 Hz
FIDRES 9.390024 Hz
AQ 0.1064960 sec
RG 188.77
DW 52.000 usec
DE 30.00 usec
TE 300.0 K
CNST2 145.0000000
CNST17 -0.5000000
D0 0.00000300 sec
D1 2.00000000 sec
D4 0.00172414 sec
D11 0.03000000 sec
D16 0.00020000 sec
D21 0.00360000 sec
D24 0.00089000 sec
IN0 0.00001660 sec
TDav 1
SFO1 800.1640008 MHz
NUC1 1H
P1 6.90 usec
P2 13.80 usec
P28 0 usec
PLW1 10.33500004 W
SFO2 201.2154821 MHz
NUC2 13C
CPDPRG[2 bi_p5m4sp_4sp.2
P3 12.00 usec
P14 500.00 usec
P24 2000.00 usec
P31 1498.00 usec
P63 1500.00 usec
PLW0 0 W
PLW2 167.57000732 W
PLW12 9.65209961 W
SPNAM[3] Crp80,0.5,20.1
SPOAL3 0.500
SPOFFS3 0 Hz
SPW3 49.15800095 W
SPNAM[7] Crp80comp.4
SPOAL7 0.500
SPOFFS7 0 Hz
SPW7 49.15800095 W
SPNAM[14 Crp48,1.5,20.2
SPOAL14 0.500
SPOFFS14 0 Hz
SPW14 23.59600067 W
SPNAM[18 Crp60_xfilt.2
SPOAL18 0.500
SPOFFS18 0 Hz
SPW18 14.21199989 W
SPNAM[31 Crp48,1.5,20.2
SPOAL31 0.500
SPOFFS31 0 Hz
SPW31 5.89890003 W
GPNAM[1] SMSQ10.100
GPZ1 80.00 %
GPNAM[2] SMSQ10.100
GPZ2 20.10 %
GPNAM[3] SMSQ10.100
GPZ3 11.00 %
GPNAM[4] SMSQ10.100
GPZ4 -5.00 %
P16 1000.00 usec
P19 600.00 usec
F1 - Acquisition parameters
TD 256
SFO1 201.2155 MHz
FIDRES 235.316269 Hz
SW 149.693 ppm
FnMODE Echo-Antiecho
F2 - Processing parameters
SI 1024
SF 800.1600142 MHz
WDW QSINE
SSB 2
LB 0 Hz
GB 0
PC 1.40
F1 - Processing parameters
SI 1024
MC2 echo-antiecho
SF 201.2003965 MHz
WDW QSINE
SSB 2
LB 0 Hz
GB 0
ppm
20
40
60
80
100
120
140
11
10
9
8
7
6
5
4
3
2
1
0
ppm

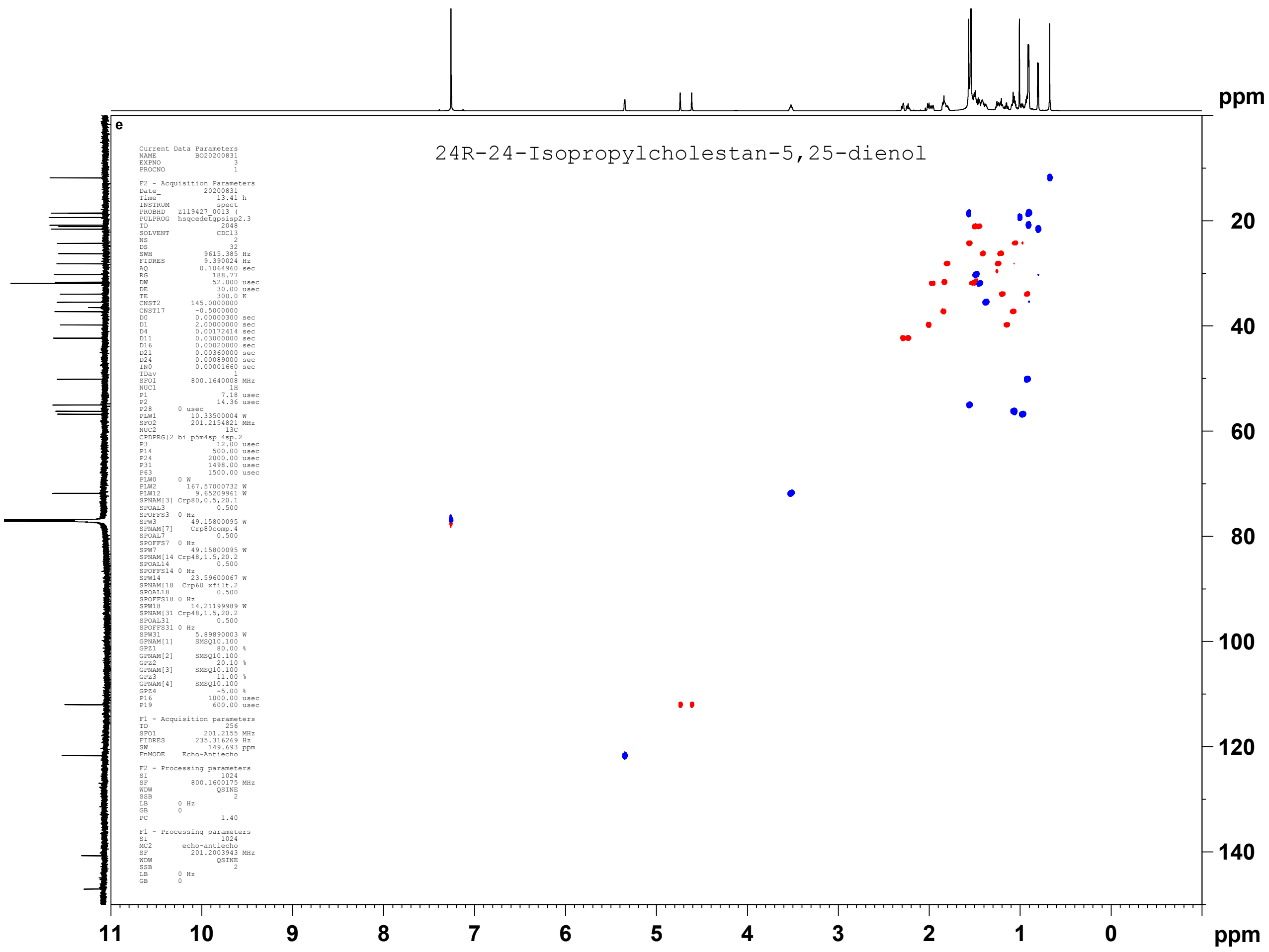
e
24R-24-Isopropylcholestan-5,25-dienol
Current Data Parameters
NAME BO20200831
EXPNO 3
PROCNO 1
F2 - Acquisition Parameters
Date_ 20200831
Time 13.41 h
INSTRUM spect
PROBHD Z119427_0013 (
PULPROG hsqcedetgpsisp2.3
TD 2048
SOLVENT CDCl3
NS 2
DS 32
SWH 9615.385 Hz
FIDRES 9.390024 Hz
AQ 0.1064960 sec
RG 188.77
DW 52.000 usec
DE 30.00 usec
TE 300.0 K
CNST2 145.0000000
CNST17 -0.5000000
D0 0.00000300 sec
D1 2.00000000 sec
D4 0.00172414 sec
D11 0.03000000 sec
D16 0.00020000 sec
D21 0.00360000 sec
D24 0.00089000 sec
IN0 0.00001660 sec
TDav 1
SFO1 800.1640008 MHz
NUC1 1H
P1 7.18 usec
P2 14.36 usec
P28 0 usec
PLW1 10.33500004 W
SFO2 201.2154821 MHz
NUC2 13C
CPDPRG[2 bi_p5m4sp_4sp.2
P3 12.00 usec
P14 500.00 usec
P24 2000.00 usec
P31 1498.00 usec
P63 1500.00 usec
PLW0 0 W
PLW2 167.57000732 W
PLW12 9.65209961 W
SPNAM[3] Crp80,0.5,20.1
SPOAL3 0.500
SPOFFS3 0 Hz
SPW3 49.15800095 W
SPNAM[7] Crp80comp.4
SPOAL7 0.500
SPOFFS7 0 Hz
SPW7 49.15800095 W
SPNAM[14 Crp48,1.5,20.2
SPOAL14 0.500
SPOFFS14 0 Hz
SPW14 23.59600067 W
SPNAM[18 Crp60_xfilt.2
SPOAL18 0.500
SPOFFS18 0 Hz
SPW18 14.21199989 W
SPNAM[31 Crp48,1.5,20.2
SPOAL31 0.500
SPOFFS31 0 Hz
SPW31 5.89890003 W
GPNAM[1] SMSQ10.100
GPZ1 80.00 %
GPNAM[2] SMSQ10.100
GPZ2 20.10 %
GPNAM[3] SMSQ10.100
GPZ3 11.00 %
GPNAM[4] SMSQ10.100
GPZ4 -5.00 %
P16 1000.00 usec
P19 600.00 usec
F1 - Acquisition parameters
TD 256
SFO1 201.2155 MHz
FIDRES 235.316269 Hz
SW 149.693 ppm
FnMODE Echo-Antiecho
F2 - Processing parameters
SI 1024
SF 800.1600175 MHz
WDW QSINE
SSB 2
LB 0 Hz
GB 0
PC 1.40
F1 - Processing parameters
SI 1024
MC2 echo-antiecho
SF 201.2003943 MHz
WDW QSINE
SSB 2
LB 0 Hz
GB 0
ppm
20
40
60
80
100
120
140
11
10
9
8
7
6
5
4
3
2
1
0
ppm

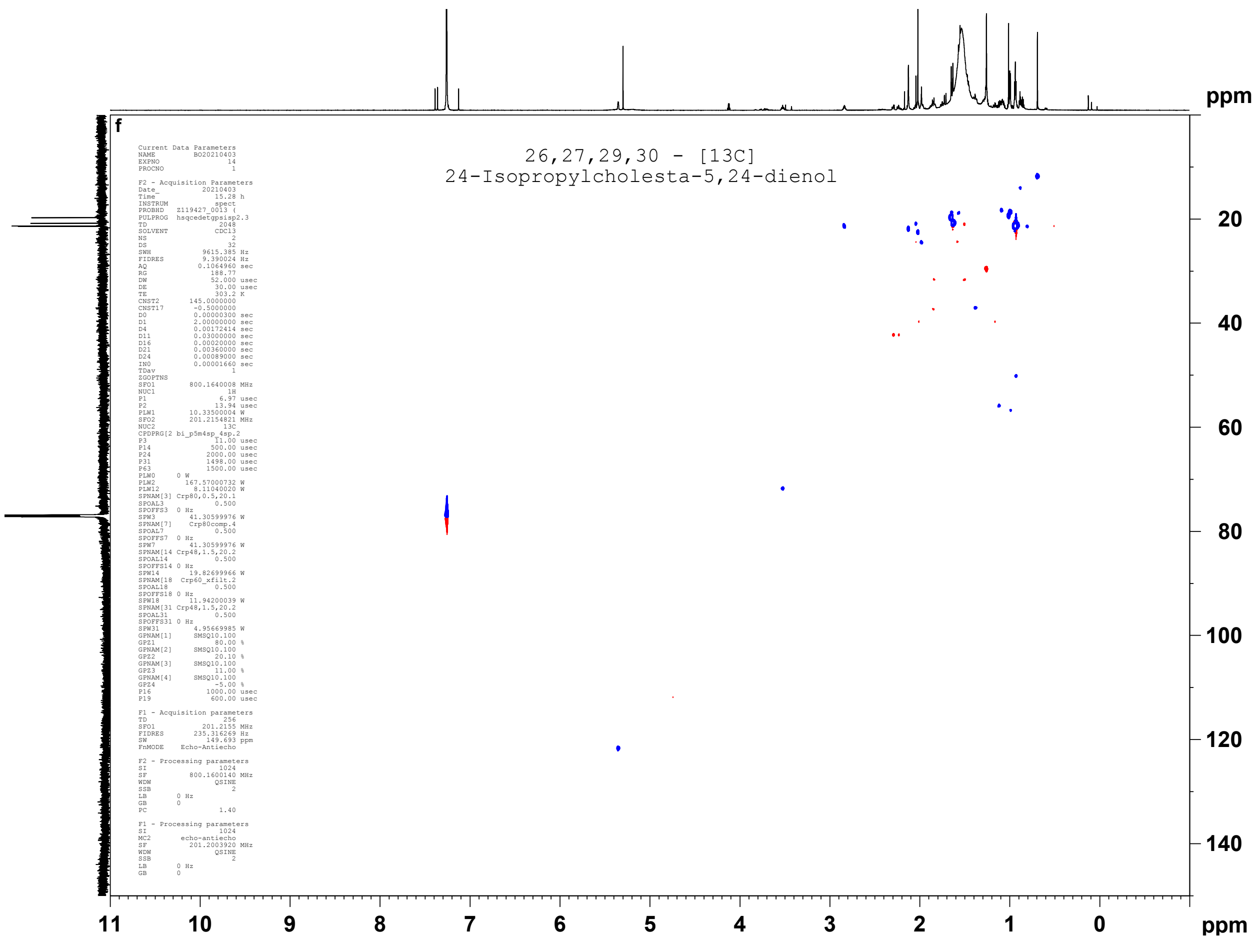
ppm
f
Current Data Parameters
NAME BO20210403
EXPNO 14
PROCNO 1
F2 - Acquisition Parameters
Date_ 20210403
Time 15.28 h
INSTRUM spect
PROBHD Z119427_0013 (
PULPROG hsqcedetgpsisp2.3
TD 2048
SOLVENT CDCl3
NS 2
DS 32
SWH 9615.385 Hz
FIDRES 9.390024 Hz
AQ 0.1064960 sec
RG 188.77
DW 52.000 usec
DE 30.00 usec
TE 303.2 K
CNST2 145.0000000
CNST17 -0.5000000
D0 0.00000300 sec
D1 2.00000000 sec
D4 0.00172414 sec
D11 0.03000000 sec
D16 0.00020000 sec
D21 0.00360000 sec
D24 0.00089000 sec
IN0 0.00001660 sec
TDav 1
ZGOPTNS
SFO1 800.1640008 MHz
NUC1 1H
P1 6.97 usec
P2 13.94 usec
PLW1 10.33500004 W
SFO2 201.2154821 MHz
NUC2 13C
CPDPRG[2 bi_p5m4sp_4sp.2
P3 11.00 usec
P14 500.00 usec
P24 2000.00 usec
P31 1498.00 usec
P63 1500.00 usec
PLW0 0 W
PLW2 167.57000732 W
PLW12 8.11040020 W
SPNAM[3] Crp80,0.5,20.1
SPOAL3 0.500
SPOFFS3 0 Hz
SPW3 41.30599976 W
SPNAM[7] Crp80comp.4
SPOAL7 0.500
SPOFFS7 0 Hz
SPW7 41.30599976 W
SPNAM[14 Crp48,1.5,20.2
SPOAL14 0.500
SPOFFS14 0 Hz
SPW14 19.82699966 W
SPNAM[18 Crp60_xfilt.2
SPOAL18 0.500
SPOFFS18 0 Hz
SPW18 11.94200039 W
SPNAM[31 Crp48,1.5,20.2
SPOAL31 0.500
SPOFFS31 0 Hz
SPW31 4.95669985 W
GPNAM[1] SMSQ10.100
GPZ1 80.00 %
GPNAM[2] SMSQ10.100
GPZ2 20.10 %
GPNAM[3] SMSQ10.100
GPZ3 11.00 %
GPNAM[4] SMSQ10.100
GPZ4 -5.00 %
P16 1000.00 usec
P19 600.00 usec
F1 - Acquisition parameters
TD 256
SFO1 201.2155 MHz
FIDRES 235.316269 Hz
SW 149.693 ppm
FnMODE Echo-Antiecho
F2 - Processing parameters
SI 1024
SF 800.1600140 MHz
WDW QSINE
SSB 2
LB 0 Hz
GB 0
PC 1.40
F1 - Processing parameters
SI 1024
MC2 echo-antiecho
SF 201.2003920 MHz
WDW QSINE
SSB 2
LB 0 Hz
GB 0
26,27,29,30 - [13C]
24-Isopropylcholesta-5,24-dienol
20
40
60
80
100
120
140
11
10
9
8
7
6
5
4
3
2
1
0
ppm

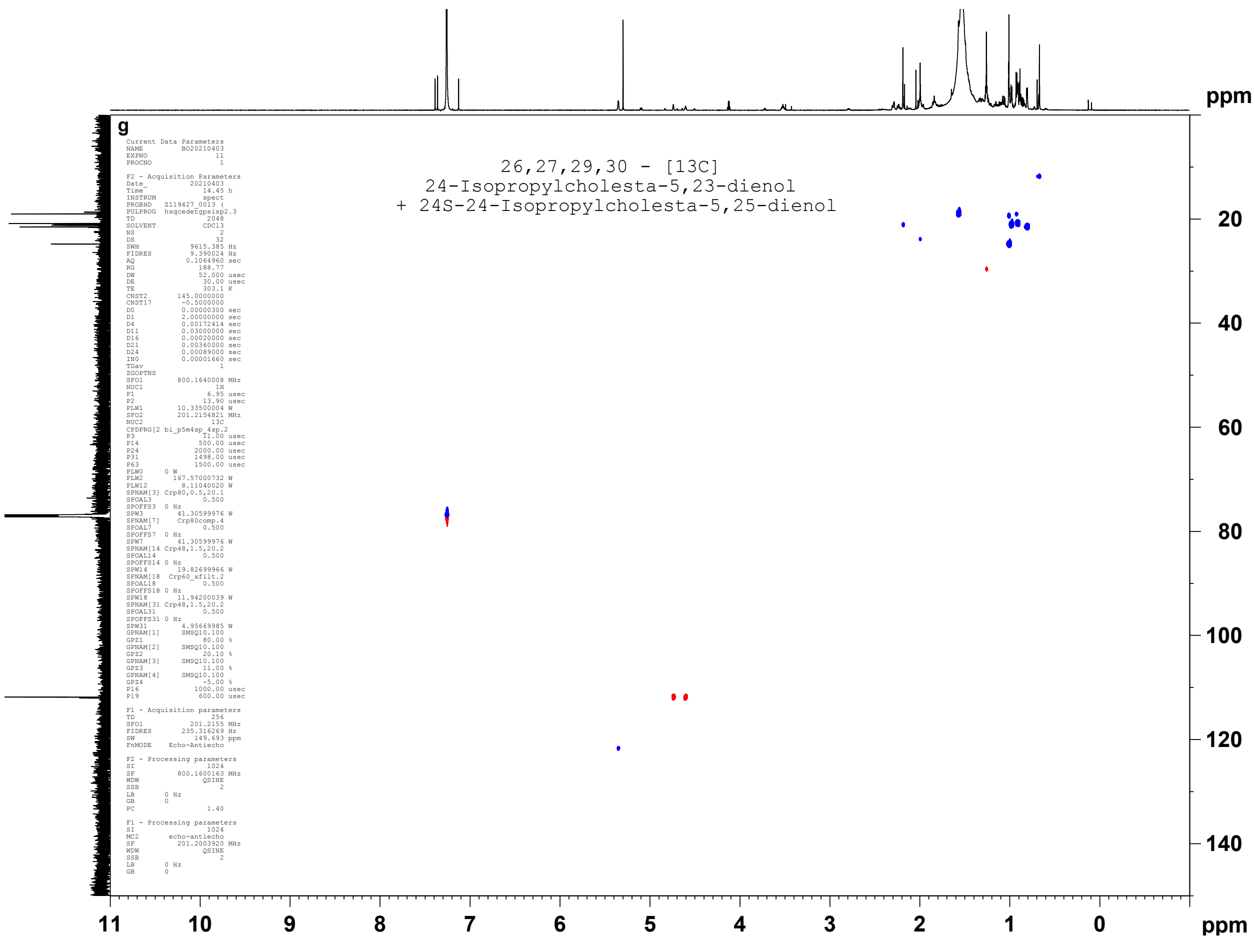
g
26,27,29,30 - [13C]
24-Isopropylcholesta-5,23-dienol
+ 24S-24-Isopropylcholesta-5,25-dienol
ppm
20
40
60
80
100
120
140
11
10
9
8
7
6
5
4
3
2
1
0
ppm

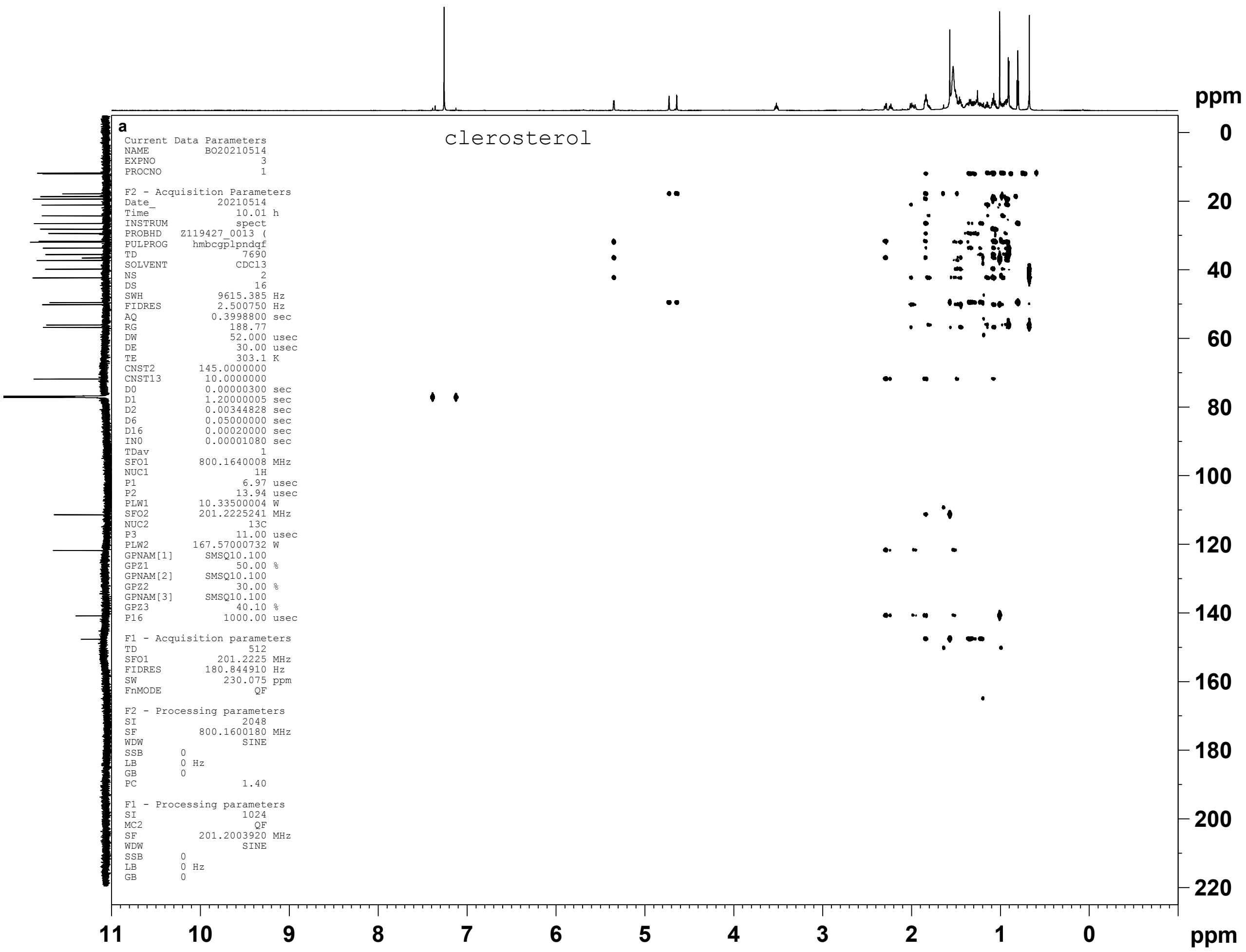


Supplementary Figure 7. **Raw 2D NMR (HMBC) spectra of the following sterols:** **a** clerosterol, **b** epiclerosterol, **c** 24*S*-24-isopropylcholesta-5,25-dienol, **d** 24*R*-24-isopropylcholesta-5,25-dienol, and **e** [26, 27, 29, 30-$^{13}$C] 24-isopropylcholesta-5,24-dienol. Chemical shifts are summarized in Supplementary Table 3.

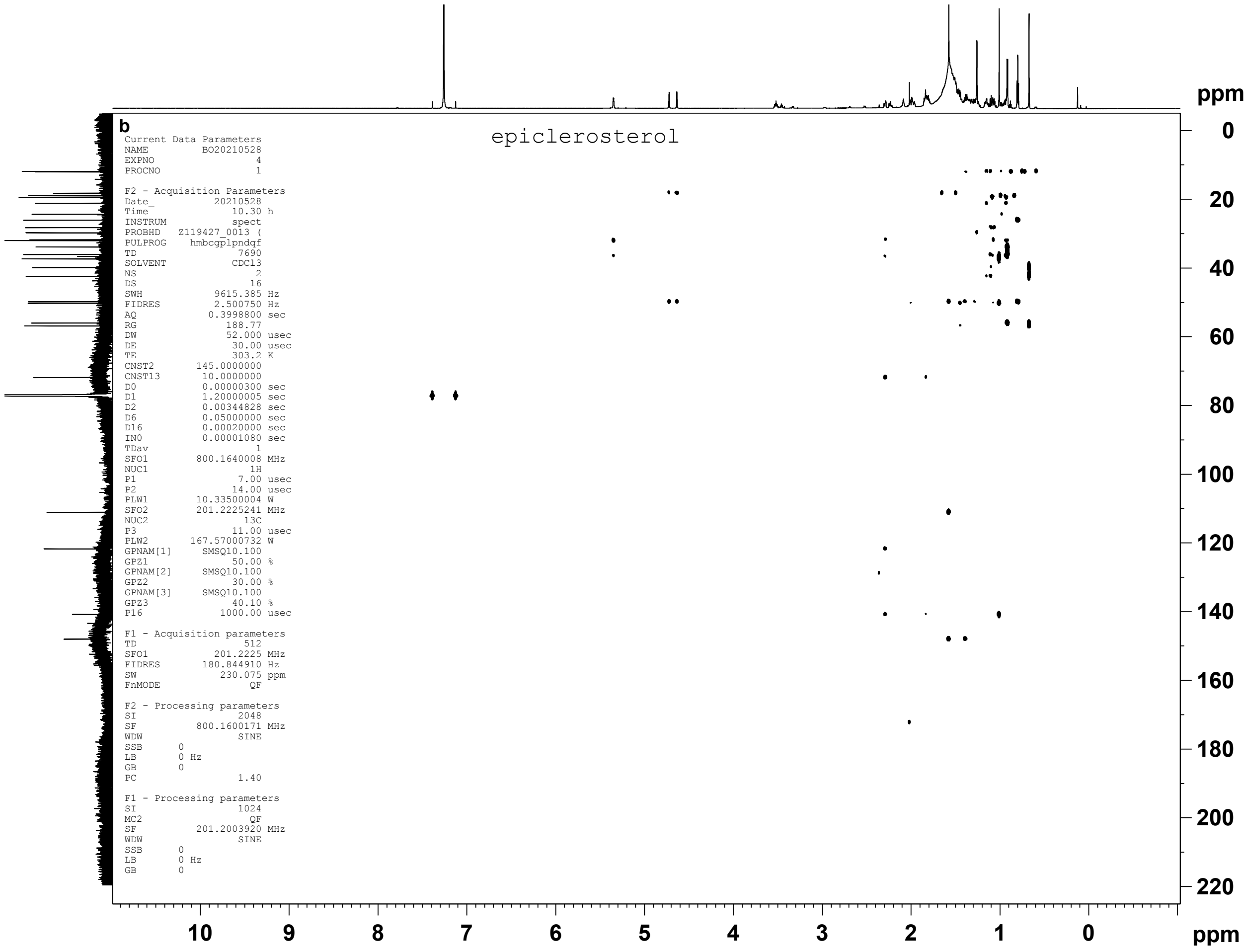

b
epiclerosterol
Current Data Parameters
NAME BO20210528
EXPNO 4
PROCNO 1
F2 - Acquisition Parameters
Date_ 20210528
Time_ 10.30 h
INSTRUM spect
PROBHD Z119427_0013 (
PULPROG hmbcgplpndqf
TD 7690
SOLVENT CDCl3
NS 2
DS 16
SWH 9615.385 Hz
FIDRES 2.500750 Hz
AQ 0.3998800 sec
RG 188.77
DW 52.000 usec
DE 30.00 usec
TE 303.2 K
CNST2 145.0000000
CNST13 10.0000000
D0 0.00000300 sec
D1 1.20000005 sec
D2 0.00344828 sec
D6 0.05000000 sec
D16 0.00020000 sec
IN0 0.00001080 sec
TDav 1
SFO1 800.1640008 MHz
NUC1 1H
P1 7.00 usec
P2 14.00 usec
PLW1 10.33500004 W
SFO2 201.2225241 MHz
NUC2 13C
P3 11.00 usec
PLW2 167.57000732 W
GPNAM[1] SMSQ10.100
GPZ1 50.00 %
GPNAM[2] SMSQ10.100
GPZ2 30.00 %
GPNAM[3] SMSQ10.100
GPZ3 40.10 %
P16 1000.00 usec
F1 - Acquisition parameters
TD 512
SFO1 201.2225 MHz
FIDRES 180.844910 Hz
SW 230.075 ppm
FnMODE QF
F2 - Processing parameters
SI 2048
SF 800.1600171 MHz
WDW SINE
SSB 0
LB 0 Hz
GB 0
PC 1.40
F1 - Processing parameters
SI 1024
MC2 QF
SF 201.2003920 MHz
WDW SINE
SSB 0
LB 0 Hz
GB 0
ppm
0
20
40
60
80
100
120
140
160
180
200
220
10
9
8
7
6
5
4
3
2
1
0
ppm

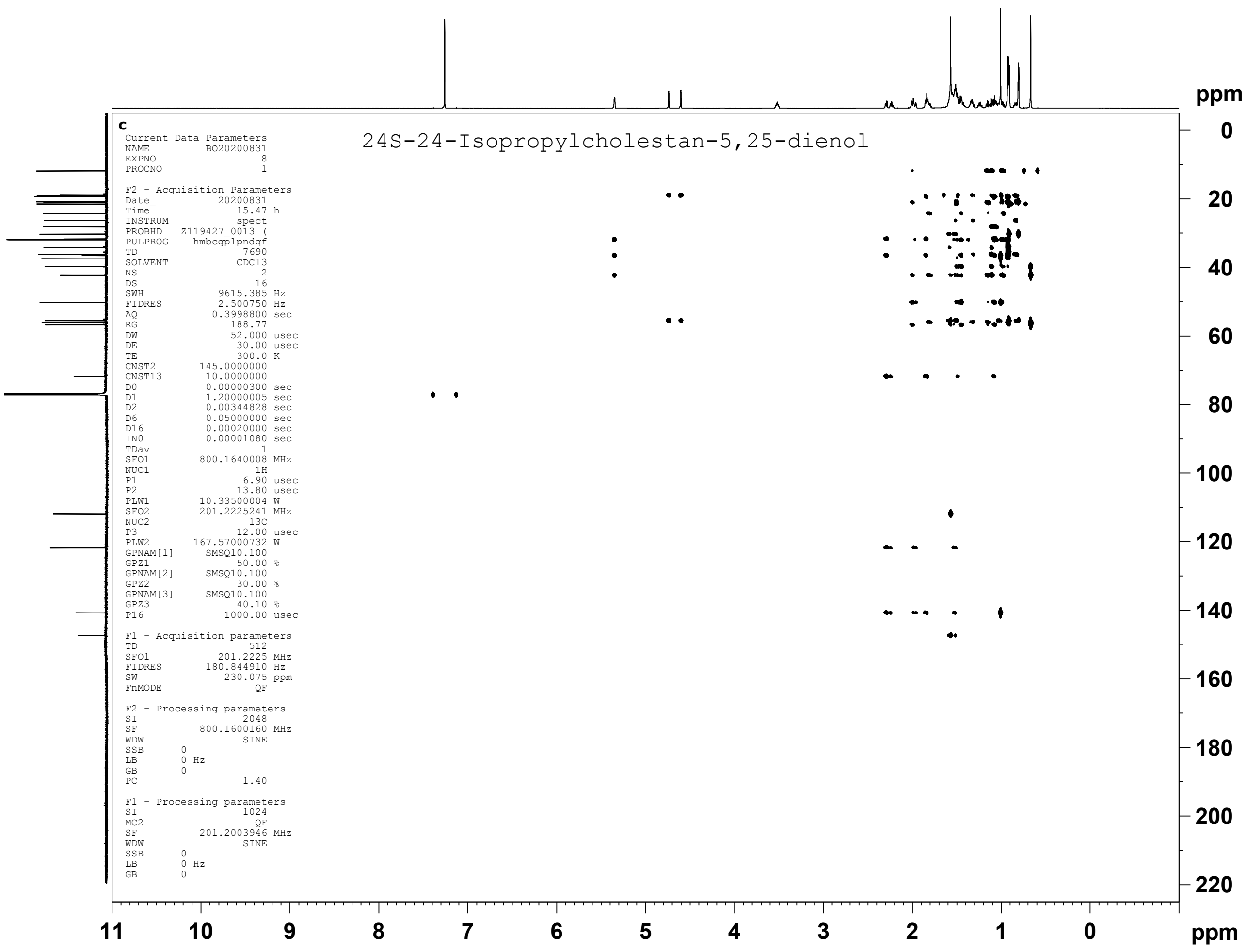
c
24S-24-Isopropylcholestan-5,25-dienol
Current Data Parameters
NAME BO20200831
EXPNO 8
PROCNO 1
F2 - Acquisition Parameters
Date_ 20200831
Time 15.47 h
INSTRUM spect
PROBHD Z119427_0013 (
PULPROG hmbcgplpndqf
TD 7690
SOLVENT CDCl3
NS 2
DS 16
SWH 9615.385 Hz
FIDRES 2.500750 Hz
AQ 0.3998800 sec
RG 188.77
DW 52.000 usec
DE 30.00 usec
TE 300.0 K
CNST2 145.0000000
CNST13 10.0000000
D0 0.00000300 sec
D1 1.20000005 sec
D2 0.00344828 sec
D6 0.05000000 sec
D16 0.00020000 sec
IN0 0.00001080 sec
TDav 1
SFO1 800.1640008 MHz
NUC1 1H
P1 6.90 usec
P2 13.80 usec
PLW1 10.33500004 W
SFO2 201.2225241 MHz
NUC2 13C
P3 12.00 usec
PLW2 167.57000732 W
GPNAM[1] SMSQ10.100
GPZ1 50.00 %
GPNAM[2] SMSQ10.100
GPZ2 30.00 %
GPNAM[3] SMSQ10.100
GPZ3 40.10 %
P16 1000.00 usec
F1 - Acquisition parameters
TD 512
SFO1 201.2225 MHz
FIDRES 180.844910 Hz
SW 230.075 ppm
FnMODE QF
F2 - Processing parameters
SI 2048
SF 800.1600160 MHz
WDW SINE
SSB 0
LB 0 Hz
GB 0
PC 1.40
F1 - Processing parameters
SI 1024
MC2 QF
SF 201.2003946 MHz
WDW SINE
SSB 0
LB 0 Hz
GB 0
ppm
0
20
40
60
80
100
120
140
160
180
200
220
11
10
9
8
7
6
5
4
3
2
1
0
ppm

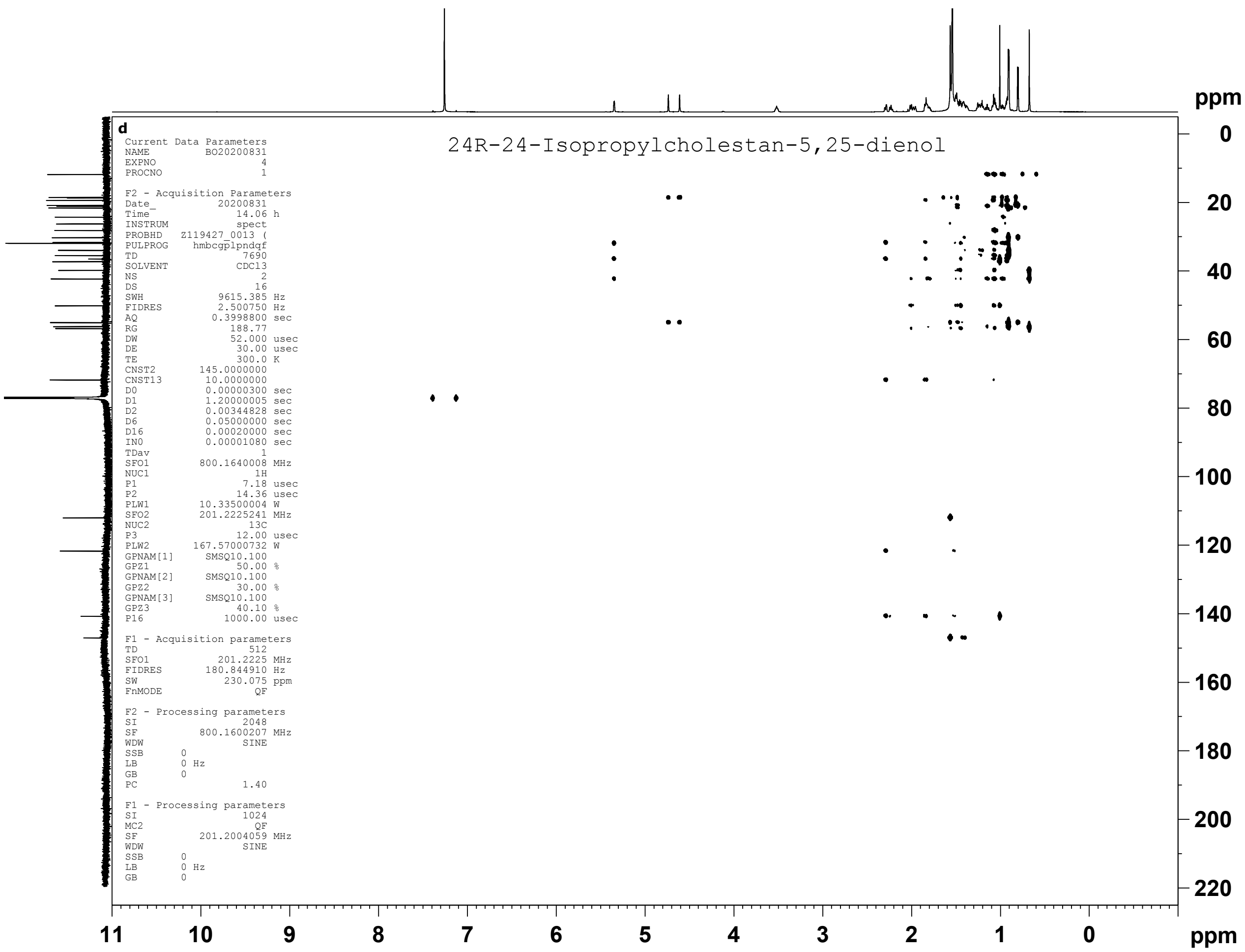
d
24R-24-Isopropylcholestan-5,25-dienol
Current Data Parameters
NAME BO20200831
EXPNO 4
PROCNO 1
F2 - Acquisition Parameters
Date_ 20200831
Time 14.06 h
INSTRUM spect
PROBHD Z119427_0013 (
PULPROG hmbcgplpndqf
TD 7690
SOLVENT CDCl3
NS 2
DS 16
SWH 9615.385 Hz
FIDRES 2.500750 Hz
AQ 0.3998800 sec
RG 188.77
DW 52.000 usec
DE 30.00 usec
TE 300.0 K
CNST2 145.0000000
CNST13 10.0000000
D0 0.00000300 sec
D1 1.20000005 sec
D2 0.00344828 sec
D6 0.05000000 sec
D16 0.00020000 sec
IN0 0.00001080 sec
TDav 1
SFO1 800.1640008 MHz
NUC1 1H
P1 7.18 usec
P2 14.36 usec
PLW1 10.33500004 W
SFO2 201.2225241 MHz
NUC2 13C
P3 12.00 usec
PLW2 167.57000732 W
GPNAM[1] SMSQ10.100
GPZ1 50.00 %
GPNAM[2] SMSQ10.100
GPZ2 30.00 %
GPNAM[3] SMSQ10.100
GPZ3 40.10 %
P16 1000.00 usec
F1 - Acquisition parameters
TD 512
SFO1 201.2225 MHz
FIDRES 180.844910 Hz
SW 230.075 ppm
FnMODE QF
F2 - Processing parameters
SI 2048
SF 800.1600207 MHz
WDW SINE
SSB 0
LB 0 Hz
GB 0
PC 1.40
F1 - Processing parameters
SI 1024
MC2 QF
SF 201.2004059 MHz
WDW SINE
SSB 0
LB 0 Hz
GB 0
ppm
0
20
40
60
80
100
120
140
160
180
200
220
11
10
9
8
7
6
5
4
3
2
1
0
ppm

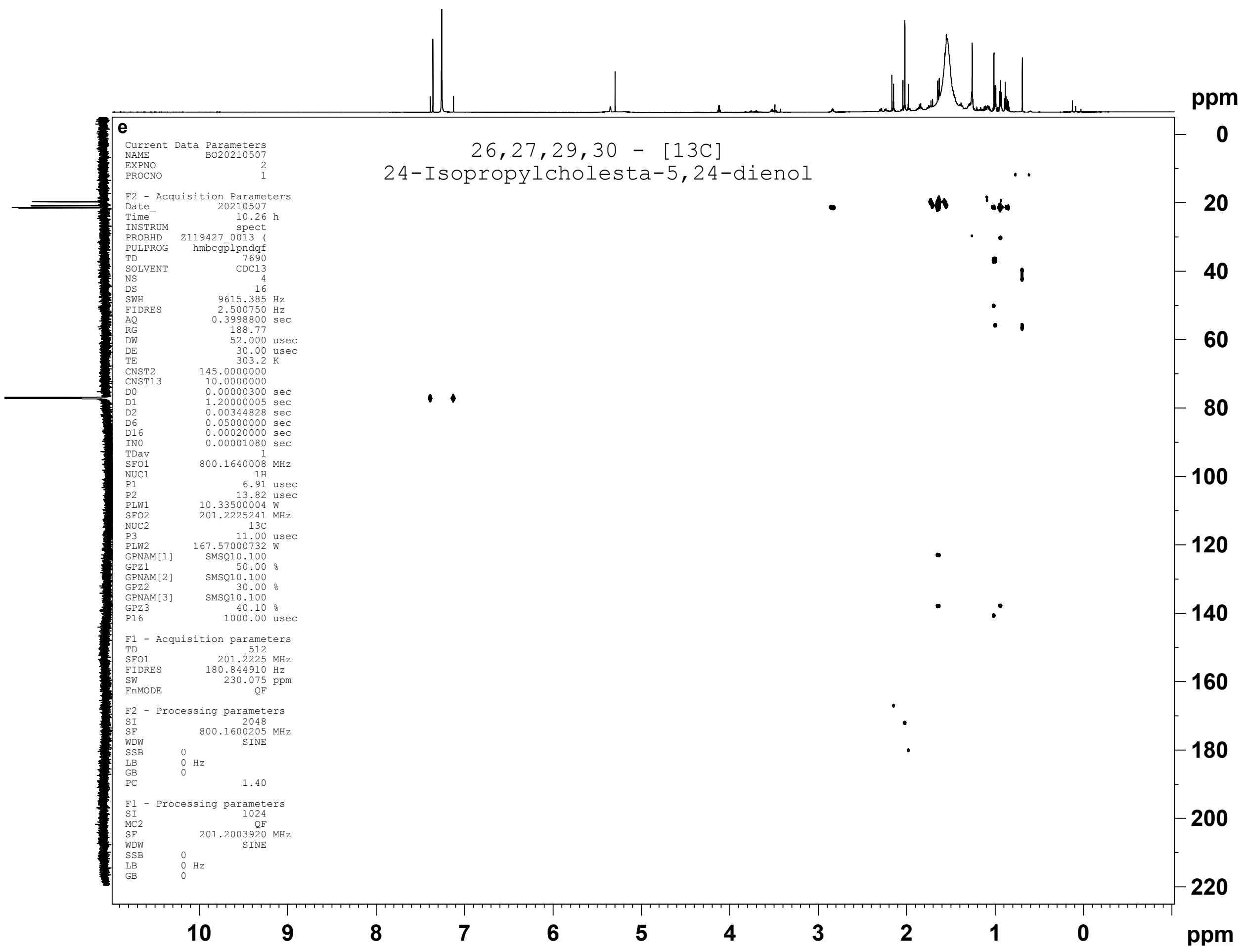
e
26,27,29,30 - [13C]
24-Isopropylcholesta-5,24-dienol
Current Data Parameters
NAME BO20210507
EXPNO 2
PROCNO 1
F2 - Acquisition Parameters
Date_ 20210507
Time 10.26 h
INSTRUM spect
PROBHD Z119427_0013 (
PULPROG hmbcgplpndqf
TD 7690
SOLVENT CDCl3
NS 4
DS 16
SWH 9615.385 Hz
FIDRES 2.500750 Hz
AQ 0.3998800 sec
RG 188.77
DW 52.000 usec
DE 30.00 usec
TE 303.2 K
CNST2 145.0000000
CNST13 10.0000000
D0 0.00000300 sec
D1 1.20000005 sec
D2 0.00344828 sec
D6 0.05000000 sec
D16 0.00020000 sec
IN0 0.00001080 sec
TDav 1
SFO1 800.1640008 MHz
NUC1 1H
P1 6.91 usec
P2 13.82 usec
PLW1 10.33500004 W
SFO2 201.2225241 MHz
NUC2 13C
P3 11.00 usec
PLW2 167.57000732 W
GPNAM[1] SMSQ10.100
GPZ1 50.00 %
GPNAM[2] SMSQ10.100
GPZ2 30.00 %
GPNAM[3] SMSQ10.100
GPZ3 40.10 %
P16 1000.00 usec
F1 - Acquisition parameters
TD 512
SFO1 201.2225 MHz
FIDRES 180.844910 Hz
SW 230.075 ppm
FnMODE QF
F2 - Processing parameters
SI 2048
SF 800.1600205 MHz
WDW SINE
SSB 0
LB 0 Hz
GB 0
PC 1.40
F1 - Processing parameters
SI 1024
MC2 QF
SF 201.2003920 MHz
WDW SINE
SSB 0
LB 0 Hz
GB 0
ppm
0
20
40
60
80
100
120
140
160
180
200
220
10
9
8
7
6
5
4
3
2
1
0
ppm

Supplementary Table 1. **Sterols previously identified in sponges relevant to this study.** The major sterol and the presence and/or percentages of $C_{27}$-$C_{30}$ sterols for each species are given when available.

| Sponge species | Major sterol | $C_{27}$ | $C_{28}$ | $C_{29}$ | $C_{30}$ | Reference |
|---|---|---|---|---|---|---|
| **DEMOSPONGIAE** | | | | | | |
| *Amphimedon queenslandica* | cholesterol | 40 | 24 | 36 | | Gold et al., 2017[1] |
| *Chondrilla nucula* | cholestanol | yes | yes | yes | | Bergmann and McTigue, 1948[2] |
| *Sarcotragus fasciculatus* | | | yes | yes | | Venkateswarlu et al., 1996[3] |
| *Haliclona amboinensis* | fucosterol | 19 | 24 | 56 | | Fromont et al., 1994[4] |
| *Aplysina aerophoba* | aplysterol | 13 | 9 | 77 | 1* | Nechev et al., 2002[5]<br>Zumberge et al., 2018[6] |
| *Petrosia ficiformis* | petrosterol | >2 | >6 | >60 | yes | Khalil and Djerassi, 1980[7] |
| *Theonella swinhoei* | theonellasterol | yes | yes | yes | | Kho 1980[8] |
| **HOMOSCLEROMORPHA** | | | | | | |
| *Oscarella lobularis* | | yes | yes | yes | | Aiello et al., 1991[9] |
| **CALCAREA** | | | | | | |
| *Sycon sp.* 1 | cholesterol | 33 | 28 | 34 | | Bergquist et al., 1986[10] |
| *Sycon sp.* 2 | cholesterol | 54 | 25 | 21 | | Bergquist et al., 1986[10] |

*Nechev et al., 2002[5] reports 1.2% 24-propyl-cholest-5,24(28)-dien-3β-ol in *A. aerophoba*. Zumberge et al., 2018[6] reports 0.5% 24-npc and 0.4% 24-ipc after catalytic hydropyrolysis of *A. aerophoba* biomass. 24-isopropyl sterols have not been reported as components of *A. aerophoba*'s native sterols, to our knowledge.

Supplementary Table 2. **Whokaryote predicts all metagenomic contigs containing sterol methyltransferases analyzed in this study as prokaryotic.**

| SMT | contig | contig length | nr genes | ratio same orientation | ID general avg | ID general std | gene density | gene length | ID Q1 | ID Q3 | rbs ratio | tiara pred | predicted |
|---|---|---|---|---|---|---|---|---|---|---|---|---|---|
| *Aplysina aerophoba* JGIcombinedJ30088_10000835 | JGIcombinedJ30088_10000835 | 32339 | 23 | 0.68 | 128.14 | 183.70 | 0.91 | 1285.09 | 3.75 | 181.00 | 0.96 | 1.0 | prokaryote |
| *Aplysina aerophoba* Ga0209021_1000146 | Ga0209021_1000146 | 97163 | 103 | 0.68 | 115.34 | 117.55 | 0.88 | 829.83 | 31.00 | 173.75 | 0.90 | 1.0 | prokaryote |
| *Petrosia ficiformis* LXNJ01014139 | LXNJ01014139.1 | 3313 | 4 | 0.00 | 100.67 | 62.53 | 0.91 | 752.00 | 56.50 | 124.50 | 0.25 | 1.0 | prokaryote |
| *Petrosia ficiformis* LXNJ01003202 | LXNJ01003202.1 | 8597 | 8 | 0.57 | 115.57 | 167.57 | 0.91 | 975.50 | 0.00 | 131.00 | 0.88 | 1.0 | prokaryote |
| *Petrosia ficiformis* LXNJ01000389 | LXNJ01000389.1 | 31336 | 22 | 0.67 | 79.90 | 78.14 | 0.94 | 1341.91 | 4.00 | 147.00 | 0.77 | 1.0 | prokaryote |
| *Petrosia ficiformis* LXNJ01004423 | LXNJ01004423.1 | 7072 | 6 | 0.60 | 102.20 | 58.00 | 0.93 | 1093.00 | 63.00 | 126.00 | 0.67 | 1.0 | prokaryote |
| *Sarcotragus foetidus* | LXNI01004480.1 | 7225 | 5 | 0.50 | 105.25 | 156.88 | 0.94 | 1359.20 | 6.75 | 121.00 | 0.80 | 1.0 | prokaryote |
| *Theonella swinhoei* associated Chromatiales sp. 1 of 2 | 2690316284 | 444506 | 427 | 0.74 | 74.66 | 122.32 | 0.93 | 968.13 | 1.00 | 104.25 | 0.68 | 1.0 | prokaryote |
| *Theonella swinhoei* associated Chromatiales sp. 2 of 2 | 2690316289 | 117771 | 97 | 0.83 | 74.25 | 116.07 | 0.94 | 1142.96 | 0.00 | 97.50 | 0.65 | 1.0 | prokaryote |
| Marine sediment, Gulf of Thailand | Ga0118733_100012602 | 21160 | 19 | 0.89 | 72.06 | 133.71 | 0.94 | 1042.53 | 0.00 | 65.50 | 0.84 | 1.0 | prokaryote |
| Marine sediment, Helgoland, North Sea | Ga0055584_100171735 | 2192 | 3 | 0.50 | 80.50 | 75.50 | 0.93 | 676.00 | 42.75 | 118.25 | 0.00 | 1.0 | prokaryote |
| Western Arctic Ocean | Ga0133547_10420286 | 2727 | 3 | 0.00 | 147.00 | 126.00 | 0.89 | 810.00 | 84.00 | 210.00 | 0.33 | 1.0 | prokaryote |
| Freshwater, Lake Fryxell, Antarctica | Ga0105048_10034691 | 7428 | 9 | 0.63 | 86.00 | 80.31 | 0.88 | 722.33 | 13.50 | 104.75 | 0.44 | 1.0 | prokaryote |
| Lake sediment, Walker Lake, Nevada | Ga0114946_10008497 | 6500 | 5 | 0.50 | 77.25 | 71.98 | 0.93 | 1212.80 | 22.50 | 103.75 | 0.40 | 1.0 | prokaryote |
| Hotspring, Beatty, Nevada | Ga0114945_10021081 | 3480 | 3 | 0.00 | 63.00 | 7.00 | 0.96 | 1108.00 | 59.50 | 66.50 | 0.33 | 1.0 | prokaryote |
| Hotspring sediment, Dewer Creek, BC | Ga0073932_1004097 | 18020 | 12 | 0.82 | 164.73 | 132.22 | 0.89 | 1342.75 | 67.00 | 269.00 | 0.92 | 1.0 | prokaryote |
| Chlamydiae sp., drinking water 1 and 2 | 2619626156 | 1146800 | 1105 | 0.68 | 85.18 | 128.28 | 0.92 | 956.10 | 0.00 | 118.00 | 0.42 | 1.0 | prokaryote |
| *Nitrospira* sp., coral reef 1 and 2 | 2681831735 | 70077 | 63 | 0.74 | 147.08 | 145.66 | 0.87 | 962.71 | 27.00 | 196.00 | 0.89 | 1.0 | prokaryote |
| *Sandaracinus* sp., marine 1 and 2 | 2775515747 | 24518 | 17 | 0.88 | 112.25 | 106.29 | 0.89 | 1282.47 | 22.50 | 163.50 | 0.76 | 1.0 | prokaryote |
| Spirochaetes sp., soil | 2708772188 | 70818 | 55 | 0.54 | 151.69 | 167.86 | 0.89 | 1142.60 | 6.25 | 242.50 | 0.24 | 1.0 | prokaryote |

Supplementary Table 3. **800 MHz $^{1}$H and 201 MHz $^{13}$C NMR assignments of selected sterol side-chain positions at 30 °C.** $^{13}$C data are in boldface. The assignments were determined by HSQC-DEPT, HMBC, and COSY 2D-NMR experiments. $^{1}$H and $^{13}$C chemical shifts are given to three and two decimal places, respectively, if directly observed, two and one, respectively, if determined from 2D spectra. Diastereotopic signals are not assigned. The raw spectra used to generate this table are provided in Supplementary Fig. 4-7.

| | C-24 | C-25 | C-26 | C-27 | C-28 | C-29 | C-30 |
|---|---|---|---|---|---|---|---|
| Fucosterol | **145.6** | 2.199<br>**34.7** | | | 5.183<br>**115.58** | 1.574<br>**13.16** | |
| Isofucosterol | **147.1** | 2.829<br>**28.6** | | | 5.108<br>**116.48** | 1.591<br>**12.76** | |
| Clerosterol* | 1.838<br>**49.54** | **147.61** | 4.730, 4.642<br>**111.33** | 1.570<br>**17.85** | 1.305, 1.341<br>**26.53** | 0.805<br>**12.04** | |
| Epiclerosterol | 1.81<br>**49.75** | **147.98** | 4.725, 4.636<br>**111.06** | 1.576<br>**18.13** | 1.290, 1.380<br>**26.01** | 0.800<br>**11.96** | |
| 24*R*-24-Isopropylcholest-5,25-dienol* | 1.56<br>**55.03** | **147.29** | 4.613, 4.738<br>**112.02** | 1.567<br>**18.63** | 1.49<br>**30.25** | 0.911<br>**20.85** | 0.803<br>**21.60** |
| 24*S*-24-Isopropylcholest-5,25-dienol | 1.52<br>**55.50** | **147.38** | 4.602 4.740<br>**111.85** | 1.570<br>**18.95** | 1.51<br>**30.24** | 0.913<br>**20.85** | 0.807<br>**21.49** |
| 24-Isopropylcholest-5,23-dienol | | | 1.013<br>**24.77** | 1.013<br>**24.79** | | 0.980<br>**20.96** | 0.980<br>**21.18** |
| 24-Isopropylcholest-5,24-dienol | **137.0** | **123.1** | 1.633<br>**20.81** | 1.651<br>**19.73** | | 0.938<br>**21.34** | 0.938<br>**21.41** |

*Clerosterol and 24*R*-24-isopropylcholest-5,25-dienol were not detected as products of the *A. aerophoba* symbiont and Chlamydiae SMTs but are shown for comparative purposes.

Supplementary Table 4. **Sources of sterol methyltransferases analyzed in this study.**

| SMT | Source | Genome ID/ BioProject | Gene ID/Locus |
|---|---|---|---|
| **DEMOSPONGIAE** | | | |
| *Amphimedon queenslandica* | JGI IMG | 2507525020 | 2508051218 |
| *Chondrilla nucula* | ([11]) | | |
| *Sarcotragus fasciculatus* | ([11]) | | |
| *Haliclona amboinensis* | Compagen | | |
| *Haliclona tubifera* | Compagen | | |
| **HOMOSCLEROMORPHA** | | | |
| *Oscarella pearsei* | Compagen | | |
| *Oscarella carmela* | Compagen | | |
| **CALCAREA** | | | |
| *Sycon ciliatum* | Compagen | | |
| **DEMOSPONGE METAGENOME** | | | |
| *Aplysina aerophoba* JGIcombinedJ30088_10000835 1 of 2 | JGI IMG | 3300002448 | JGIcombinedJ30088_1000083511 |
| *Aplysina aerophoba* JGIcombinedJ30088_10000835 2 of 2 | JGI IMG | 3300002448 | JGIcombinedJ30088_100008359 |
| *Aplysina aerophoba* Ga0209021_1000146 | JGI IMG | 3300027386 | Ga0209021_100014628 |
| *Petrosia ficiformis* LXNJ01014139 | GenBank | PRJNA318959 | LXNJ01014139.1 |
| *Petrosia ficiformis* LXNJ01003202 | GenBank | PRJNA318959 | LXNJ01003202.1 |
| *Petrosia ficiformis* LXNJ01000389 | GenBank | PRJNA318959 | LXNJ01000389.1 |
| *Petrosia ficiformis* LXNJ01004423 | GenBank | PRJNA318959 | LXNJ01004423.1 |
| *Sarcotragus foetidus* | GenBank | PRJNA318959 | LXNI01004480.1 |
| **DEMOSPONGE METAGENOME-ASSEMBLED GENOME** | | | |
| *Theonella swinhoei* associated Chromatiales sp. 1 of 2 | JGI IMG | 2690315631 | 2690455922 |
| *Theonella swinhoei* associated Chromatiales sp. 2 of 2 | JGI IMG | 2690315631 | 2690456335 |
| **OTHER METAGENOME** | | | |
| Marine sediment, Gulf of Thailand | JGI IMG | 3300010430 | Ga0118733_1000126028 |
| Marine sediment, Helgoland, North Sea | JGI IMG | 3300004097 | Ga0055584_1001717352 |
| Western Arctic Ocean | JGI IMG | 3300010883 | Ga0133547_104202862 |
| Freshwater, Lake Fryxell, Antarctica | JGI IMG | 3300009032 | Ga0105048_100346915 |
| Lake sediment, Walker Lake, Nevada | JGI IMG | 3300009504 | Ga0114946_100084975 |
| Hot spring, Beatty, Nevada | JGI IMG | 3300009444 | Ga0114945_100210811 |
| Hot spring sediment, Dewer Creek, BC | JGI IMG | 3300007072 | Ga0073932_10040971 |
| **OTHER METAGENOME-ASSEMBLED GENOME** | | | |
| Chlamydiae sp., drinking water 1 of 2 | JGI IMG | 2619618861 | 2619789262 |
| Chlamydiae sp., drinking water 2 of 2 | JGI IMG | 2619618861 | 2619789261 |
| *Nitrospira* sp., coral reef 1 of 2 | JGI IMG | 2681812944 | 2682229274 |
| *Nitrospira* sp., coral reef 2 of 2 | JGI IMG | 2681812944 | 2682229270 |
| *Sandaracinus* sp., marine 1 of 2 | JGI IMG | 2775506781 | 2775843484 |
| *Sandaracinus* sp., marine 2 of 2 | JGI IMG | 2775506781 | 2775843485 |
| Spirochaetes sp., soil | JGI IMG | 2708742746 | 2709604172 |

Supplementary Table 5. **Best amino acid substitution models, gamma shape parameters, and invariable sites proportions under the Bayesian Information Criterion and Akaike Information Criterion using ModelTest-NG on XSEDE for generation of sterol methyltransferase and oxidosqualene cyclase maximum-likelihood trees.**

| Protein | Substitution Model | Gamma Shape Parameter | Invariable Sites Proportion |
|---|---|---|---|
| sterol methyltransferase | LG+I+G | 1.260 | 0.020 |
| oxidosqualene cyclase | LG+I+G | 1.212 | 0.022 |

## SUPPLEMENTARY REFERENCES


1. Gold, D. A., O'Reilly, S. S., Watson, J., Degnan, B. M., Degnan, S. M., Krömer, J. O. & Summons, R. E. Lipidomics of the sea sponge *Amphimedon queenslandica* and implication for biomarker geochemistry. *Geobiology* **15**, 836–843 (2017).
2. Bergmann, W. & McTigue, F. H. Contributions to the study of marine products. XXI. Chondrillasterol. *J Org Chem* **13**, 738–741 (1948).
3. Venkateswarlu, Y., Reddy, M. V. R. & Rao, M. R. A new epoxy sterol from the sponge *Ircinia fasciculata*. *J Nat Prod* **59**, 876–877 (1996).
4. Fromont, J., Kerr, S., Kerr, R., Riddle, M. & Murphy, P. Chemotaxonomic relationships within, and comparisons between, the orders Haplosclerida and Petrosida (Porifera: Demospongiae) using sterol complements. *Biochem Syst Ecol* **22**, 735–752 (1994).
5. Nechev, J., Christie, W. W., Robaina, R., de Diego, F., Popov, S. & Stefanov, K. Lipid composition of the sponge *Verongia aerophoba* from the Canary Islands. *Eur J Lipid Sci Technol* **104**, 800–807 (2002).
6. Zumberge, J. A., Love, G. D., Cárdenas, P., Sperling, E. A., Gunasekera, S., Rohrssen, M., Grosjean, E., Grotzinger, J. P. & Summons, R. E. Demosponge steroid biomarker 26-methylstigmastane provides evidence for Neoproterozoic animals. *Nat Ecol Evol* **2**, 1709–1714 (2018).
7. Khalil, M. W. & Djerassi, C. Minor and trace sterols in marine invertebrates XVII. (24R)-24,26-dimethylcholesta-5,26-dien-3β-ol, a new sterol from the sponge *Petrosia ficiformis*. *Steroids* **35**, 707–719 (1980).
8. Kho, E., Imagawa, D. K., Rohmer, M., Kashman, Y. & Djerassi, C. Sterols in marine invertebrates. 22. Isolation and structure elucidation of conicasterol and theonellasterol, two new 4-methylene sterols from the Red Sea sponges *Theonella conica* and *Theonella swinhoei*. *J Org Chem* **46**, 1836–1839 (1981).
9. Aiello, A., Fattorusso, E., Magno, S. & Menna, M. Isolation of five new 5α-hydroxy-6-keto-Δ7 sterols from the marine sponge *Oscarella lobularis*. *Steroids* **56**, 337–340 (1991).
10. Bergquist, P. R., Lavis, A. & Cambie, R. C. Sterol composition and classification of the porifera. *Biochem Syst Ecol* **14**, 105–112 (1986).
11. Gold, D. A., Grabenstatter, J., de Mendoza, A., Riesgo, A., Ruiz-Trillo, I. & Summons, R. E. Sterol and genomic analyses validate the sponge biomarker hypothesis. *Proc Natl Acad Sci U S A* **113**, 2684–2689 (2016).
