# Supplementary material for: Sterol methyltransferases in uncultured bacteria complicate eukaryotic biomarker interpretations
Source: Nat Commun. 2023 Apr 3;14:1859. doi: 10.1038/s41467-023-37552-3 (PMC10070321; doi:10.1038/s41467-023-37552-3)
Supplement: Supplementary file 3 — Description of Additional Supplementary Files [file 41467_2023_37552_MOESM3_ESM.pdf]

Description of Additional Supplementary Files

File Name: Supplementary Data 1
Description: JGI IMG Gene IDs, e-values, and percent identities for sterol biosynthesis homologs shown in Figure 3.

File Name: Supplementary Data 2
Description: Raw EIC data used to generate the chromatograms in Figure 1.

File Name: Supplementary Data 3
Description: Raw mass spectrometry data used to generate the spectra in Supplementary Figure 3.
