# Supplementary material for: Sterol methyltransferases in uncultured bacteria complicate eukaryotic biomarker interpretations
Source: Nat Commun. 2023 Apr 3;14:1859. doi: 10.1038/s41467-023-37552-3 (PMC10070321; doi:10.1038/s41467-023-37552-3)
Supplement: Supplementary file 7 — Reporting Summary [file 41467_2023_37552_MOESM7_ESM.pdf]

nature portfolio

Corresponding author(s): Paula Welander

Last updated by author(s): Feb 10, 2023

Reporting Summary

Nature Portfolio wishes to improve the reproducibility of the work that we publish. This form provides structure for consistency and transparency in reporting. For further information on Nature Portfolio policies, see our Editorial Policies and the Editorial Policy Checklist.

Statistics

For all statistical analyses, confirm that the following items are present in the figure legend, table legend, main text, or Methods section.

n/a	Confirmed	
☒	☐	The exact sample size (n) for each experimental group/condition, given as a discrete number and unit of measurement
☒	☐	A statement on whether measurements were taken from distinct samples or whether the same sample was measured repeatedly
☒	☐	The statistical test(s) used AND whether they are one- or two-sided<br>*Only common tests should be described solely by name; describe more complex techniques in the Methods section.*
☒	☐	A description of all covariates tested
☒	☐	A description of any assumptions or corrections, such as tests of normality and adjustment for multiple comparisons
☒	☐	A full description of the statistical parameters including central tendency (e.g. means) or other basic estimates (e.g. regression coefficient) AND variation (e.g. standard deviation) or associated estimates of uncertainty (e.g. confidence intervals)
☒	☐	For null hypothesis testing, the test statistic (e.g. F, t, r) with confidence intervals, effect sizes, degrees of freedom and P value noted<br>*Give P values as exact values whenever suitable.*
☒	☐	For Bayesian analysis, information on the choice of priors and Markov chain Monte Carlo settings
☒	☐	For hierarchical and complex designs, identification of the appropriate level for tests and full reporting of outcomes
☒	☐	Estimates of effect sizes (e.g. Cohen's d, Pearson's r), indicating how they were calculated

Our web collection on statistics for biologists contains articles on many of the points above.

Software and code

Policy information about availability of computer code

Data collection	No code was used for data collection in this study.
Data analysis	Agilent MassHunter Qualitative Analysis B.06.00, Whokaryote v0.0.1, Geneious Prime v2021.0.3, MUSCLE v3.8.1551, ModelTest-NG on XSEDE v0.1.5, IQ-Tree on XSEDE v2.1.2, HmmerWeb v2.41.1, iTOL v. 6.7.

For manuscripts utilizing custom algorithms or software that are central to the research but not yet described in published literature, software must be made available to editors and reviewers. We strongly encourage code deposition in a community repository (e.g. GitHub). See the Nature Portfolio guidelines for submitting code & software for further information.

Data

Policy information about availability of data

All manuscripts must include a data availability statement. This statement should provide the following information, where applicable:

- Accession codes, unique identifiers, or web links for publicly available datasets
- A description of any restrictions on data availability
- For clinical datasets or third party data, please ensure that the statement adheres to our policy

The data that support the findings of this study are available within this paper and its supplementary information. GenBank accession codes and JGI IMG gene IDs for analyzed metagenomic SMTs are provided in Supplementary Table 4. JGI IMG gene IDs for the additional sterol biosynthesis homologs shown in Figure 2 are provided in Supplementary Data File 1. The raw extracted ion chromatogram data used to generate Figure 1 are provided in Supplementary Data File 2. The raw mass spectrometry data used to generate Supplementary Figure 3 are provided in Supplementary Data File 3. The NMR spectra used to generate Supplementary

Table 3 are provided in Supplementary Data File 4.

Human research participants

Policy information about studies involving human research participants and Sex and Gender in Research.

Reporting on sex and gender	There were no human research participants in this study.
Population characteristics	There were no human research participants in this study.
Recruitment	There were no human research participants in this study.
Ethics oversight	There were no human research participants in this study.

Note that full information on the approval of the study protocol must also be provided in the manuscript.

Field-specific reporting

Please select the one below that is the best fit for your research. If you are not sure, read the appropriate sections before making your selection.

☒ Life sciences ☐ Behavioural & social sciences ☐ Ecological, evolutionary & environmental sciences

For a reference copy of the document with all sections, see nature.com/documents/nr-reporting-summary-flat.pdf

Life sciences study design

All studies must disclose on these points even when the disclosure is negative.

Sample size	No sample size calculation was performed as comparison between empty plasmid control strains and SMT-containing plasmid strains is sufficient to determine protein function. The number of metagenomic SMTs chosen for in vitro analysis was based on 1) coverage of multiple environments and 2) funding available for artificial synthesis.
Data exclusions	No data were excluded, we report products (or lack thereof) of each SMT we tested and include all SMTs we tested in our bioinformatics analyses.
Replication	In vitro reactions and GC-MS analyses for each SMT were performed twice for both substrates and the same products were observed each time.
Randomization	This is not relevant to this study as there were no experimental groups.
Blinding	This is not relevant to this study as there was no group allocation.

Reporting for specific materials, systems and methods

We require information from authors about some types of materials, experimental systems and methods used in many studies. Here, indicate whether each material, system or method listed is relevant to your study. If you are not sure if a list item applies to your research, read the appropriate section before selecting a response.

Materials & experimental systems

n/a	Involved in the study	
☒	☐	Antibodies
☒	☐	Eukaryotic cell lines
☒	☐	Palaeontology and archaeology
☒	☐	Animals and other organisms
☒	☐	Clinical data
☒	☐	Dual use research of concern

Methods

n/a	Involved in the study	
☒	☐	ChIP-seq
☒	☐	Flow cytometry
☒	☐	MRI-based neuroimaging
